# Contexts and Mechanisms Related to the Efficacy of Digital Medication Adherence Interventions to Support Medication Adherence Among Adults With Chronic Diseases: Realist Review

**DOI:** 10.2196/89803

**Published:** 2026-08-28

**Authors:** Srinjaya Saha, Yvonne Latham, Carol Holland

**Affiliations:** 1 Department of Health Research, Faculty of Health and Medicine, Lancaster University, Health Innovation Campus, John Fisher Drive, Bailrigg, Lancaster, England, LA1 4YW, United Kingdom, 44 01524 65201; 2 Department of Organisation, Work and Technology, Lancaster University Management School, Lancaster University, Bailrigg, Lancaster, England, United Kingdom; 3 Department of Health Research, Faculty of Health and Medicine, Lancaster University, Bailrigg, Lancaster, England, United Kingdom

**Keywords:** medication adherence, unintentional nonadherence, digital interventions, realist review, program theories

## Abstract

**Background:**

Digital medication adherence interventions for individuals diagnosed with chronic conditions have been developed, but their long-term efficacy in improving medication adherence has been limited. Exploring mechanisms and contexts related to outcomes of digital medication adherence interventions is important for these to be effectively tailored for different populations and contexts. A realist review was conducted to assess which intervention components might work for whom, and under what circumstances.

**Methods:**

A realist review of literature published between 2002‐2024 was conducted. Studies reporting digital medication adherence interventions for people diagnosed with chronic diseases and experiencing unintentional medication nonadherence issues were included. MEDLINE, CINAHL, PsycInfo, Web of Science, Scopus, Embase, and gray literature databases, Overton, and Policy Commons were searched. Data regarding contexts, mechanisms, and outcomes were extracted and synthesized into program theories. Program theories from the realist review were triangulated with findings from two rounds of workshops (three workshops in each round), in which context-mechanism-outcome (CMO) configurations were cocreated with various stakeholders of a project aimed at implementing a digital medication adherence intervention to reduce unintentional nonadherence among people with chronic diseases. There were 12 participants in each round.

**Results:**

The analysis of 83 papers and content of the cocreation workshops led to 35 CMOs theories in seven theory areas, which were (1) building and maintaining medication intake habits; (2) solving medication-related barriers to adherence; (3) enabling collection of prescription medications on time; (4) supporting people with unpredictable health conditions and sensory or motor impairments; (5) supporting individuals with high anxiety, low medication self-efficacy, and low social support; (6) supporting individuals who are uncomfortable with technology; and (7) economic, policy, and organizational factors affecting implementation of smart medication devices. Fourteen CMOs came from both the literature and workshops, 7 CMOs from the literature alone, and 14 CMOs from the workshops alone.

**Conclusions:**

Analysis of the program theories suggested a range of intervention components considering contextual factors that may improve medication adherence of individuals. These were used to form recommendations for intervention developers and implementers. Further studies are required on policy and economic factors affecting large-scale implementation of digital medication adherence interventions in different settings. Future interventions should report intervention content and delivery in detail and codevelop effective implementation strategies with professionals who can support the implementation of the intervention.

## Introduction

Medication adherence is defined as the extent to which individuals’ medication intake is consistent with their health care providers’ recommendations [1]. Medication nonadherence is particularly common among individuals diagnosed with long-term health conditions. Consequences of medication nonadherence include worsening of disease symptoms, increased rate of hospitalization, and mortality [2,3].

Medication adherence is a complex phenomenon, and nonadherence occurs in a range of different scenarios: if individuals do not start taking their prescribed medications (primary nonadherence) to begin with; take wrong doses at the wrong time; skip doses (nonconforming); or stop taking the medications against health care providers’ recommendations (nonpersistence) [4,5]. Medication nonadherence could be intentional, whereby an individual actively decides not to take medications as prescribed due to factors such as severe side-effects, or being nonintentional, which is an unplanned behavior possibly because of forgetfulness [1]. The barriers to medication adherence fall under 5 dimensions [2]: social and economic factors (eg, low socioeconomic status); health system/health care related factors (eg, patient-provider relationship); therapy related factors (eg, complexity of medication regimen); condition-related factors (eg, severity of symptoms and comorbidities); and patient related factors (eg, patient’s beliefs about perceived need for medications). It has been recommended that personalized interventions that target patients’ barriers to medication adherence in all 5 dimensions can help improve medication adherence [2].

However, the findings of a systematic review by Yap et al [6] highlighted the conflicts in evidence regarding the factors associated with medication adherence and showed that barriers to medication adherence could vary among different contexts. This highlights the importance of understanding how contextual factors may influence adherence and designing tailored interventions for specific contexts [7,8].

Digital interventions have been developed to improve medication adherence, such as providing text message reminders to take medications [8,9]. Digital interventions have the potential to contribute to more equitable delivery of care, helping improve health care for patients living in remote or rural areas and facilitating more frequent communication with patients; for example, medication reminders can be sent more frequently through digital interventions [10]. However, systematic reviews have highlighted the lack of evidence of long-term efficacy of digital medication adherence interventions in improving medication adherence among individuals with chronic illnesses. Interventions tailored to individual patients, targeting multiple dimensions of medication nonadherence [1,8] as recommended by the World Health Organization [2], are also lacking.

Systematic reviews have also highlighted the difficulties in understanding the mechanisms by which the interventions may have led to improvement in medication adherence, as the studies included were heterogeneous in terms of populations and content and delivery of the interventions [10,11]. Additionally, systematic and scoping reviews provide information about the efficacy or inefficacy of interventions but rarely provide a detailed analysis of contexts and mechanisms related to intervention efficacy or inefficacy [12]. To advance understanding, we need to study such contexts and mechanisms related to outcomes of digital medication adherence interventions.

To enable this understanding, we used realist review methods. A realist review aims to understand the mechanisms of *how* complex interventions work or do not work in different contexts as opposed to just *whether* they work or not [13]. Contexts (circumstances or conditions in which mechanisms can work and lead to the outcomes), mechanisms (elements of the interventions which can potentially lead to the outcomes), and outcomes are presented together as program theories to explain how interventions work [13]. As opposed to systematic reviews, realist reviews use a wider range of evidence, including gray literature, to understand what works for whom and under what conditions [12,13]. Thus, this study aimed to explore the contexts and mechanisms related to the efficacy or inefficacy of digital medication adherence interventions in improving medication adherence among patients with chronic illnesses experiencing unintentional medication adherence issues. To the best of the authors’ knowledge, this is the first realist review to assess contexts and mechanisms related to outcomes of digital medication adherence interventions and provide recommendations for designing and implementing similar digital medication interventions.

## Methods

### Overview

This study was preregistered on PROSPERO (International Prospective Register of Systematic Reviews; CRD42025641383) and has been reported according to the RAMESES (Realist and Meta-Narrative Evidence Syntheses: Evolving Standards) [14].

The glossary of terms used to describe the steps undertaken to carry out this review has been presented in Table 1.

**Table 1.** Glossary of terms used to describe the process of this realist review.

Terms	Description
Contexts	Conditions in which the intervention operates, including factors related to individuals receiving the intervention and social, cultural, economic, or policy level factors [13,15].
Mechanisms	The underlying processes that make the intervention work, which operate in certain contexts/situations to lead to outcomes. These can be classified as “mechanism resources,” the resources provided by the interventions, and “mechanism responses,” the responses that the intervention resources elicit among participants of the intervention [16].
Outcomes	The intended and/or unintended results of an intervention [14].
CMO^a^ configurations	A CMO configuration is a heuristic used in realist research to show patterns of how an intervention generates outcomes and the mechanisms that operate in contexts associated with them [14].
Program	Interventions or services under investigation [17]. In this realist review, the program refers to the digital interventions that aimed to improve medication adherence or reduce unintentional nonadherence.
Program theories	Explanations about how specific interventions work. CMOs represent individual program theories. This realist review also presents overarching program theories, which refer to the integration of individual CMO configurations into explanatory models.
Initial program theories	Initial ideas regarding the program theories of an intervention. These CMO configurations are further refined during the realist review process [15].

a CMO: context-mechanism-outcome.

The following 5 steps were taken to conduct the realist review.

### Development of Initial Program Theory

Initial program theories were cocreated in workshops with the stakeholders of a pilot project that aimed to explore the efficacy of a smart medication device called the “Medybox” in improving medication adherence among domiciliary care recipients and hospital discharge patients in a region in Northern England, United Kingdom. In the workshops, the stakeholders hypothetically discussed the features of the Medybox (a modified electronic pillbox) as an example of a digital medication adherence intervention, which informed the realist review that explored the program theories of all types of digital medication adherence interventions to reduce unintentional medication nonadherence. These stakeholders were presented with a summary of the features of the device and its implementation process, summarized as follows:

- Potential recipients of this device were adults diagnosed with any chronic disease who were unintentionally nonadherent to their medication regimens.
- Domiciliary care providers, short-term re-enablement services, and hospital discharge teams were to identify individuals who needed support to improve their medication adherence and refer them to use the device.
- The Medybox device was a modified electronic pillbox in that the device did not store medications according to individual doses. Instead, it safely stored all types of medications (except injectables and medications that needed refrigeration), including larger liquid medications. The device provided light and sound reminders for medication intake and transmitted real-time responses to the reminders (opening of the device by the participants), allowing the device providers to remotely monitor individuals’ medication adherence and provide feedback on it. The device had a built-in SIM card and did not require a Wi-Fi connection. It was to be provided to individuals for 6‐12 weeks, and during this time the device was intended to help individuals form effective habits of taking their medicines correctly and independently, after which it would be taken away. After that, the device providers planned to recommend other types of interventions or technologies to maintain recipients’ medication adherence, such as mobile apps, with a return to caregiver support as one option.

Six cocreation workshops of approximately 2 hours each in 2 workshop rounds (3 workshops and 12 participants in each round) were conducted to generate ideas for program theories for the implementation of this particular device. Stakeholders included members of the regional National Health Service integrated care board (ICB) who were former health care professionals (nurses) but currently working on a digitizing adult social care program, device providers who were implementing the intervention, ICB pharmacists, managers of domiciliary care organizations, individuals receiving care for their medication intake and their informal or family caregivers. Most of the stakeholders were women (10/12, 83%) and 83% (10/12) were White British (consistent with the ethnicity of the population in the local area).

In keeping with qualitative methods, a small number of appropriate stakeholders were recruited purposively via word of mouth and contacts of the research team, the ICB, and the device providers. Stakeholders were “experts by experience” as they had lived experiences of receiving or providing care to individuals requiring help with medication adherence. ICB professionals were also knowledgeable about possible contextual factors that could lead to unintended outcomes of the program [13,18]. The same group of professionals and care recipients were invited to participate in the second round of workshops. Additionally, participants were recruited if a certain group of stakeholders was underrepresented in the first round. One of the device providers involved in the implementation of the program dropped out after the first round. Therefore, the researchers recruited an ICB pharmacist in the second round to maintain numbers and improve diversity of the stakeholders involved in the workshops (see Saha et al [19] for detailed information about cocreation workshops). The workshops did not include academic researchers as stakeholders.

The discussions were facilitated by the research team, who provided participants with prompts regarding potential contexts (eg, technological literacy of individuals), mechanisms (medication intake reminders), and outcomes (eg, medication adherence) that were derived from the emerging findings of this review. Specific parts of the workshop discussions were audio- or audio-and-video-recorded, and the research team made notes of the workshop discussions. The context-mechanism-outcomes (CMOs) derived from the first round of workshops were presented to the participants in the second round of workshops to further refine them. A list of initial ideas of CMOs derived from workshop discussions was triangulated with the findings from the realist review. This was an iterative process in which the workshop discussions informed the realist review and vice versa. Further details about the device and the prompts used to facilitate workshop discussions are presented in Multimedia Appendix 1. This method of conducting cocreation workshops has been used to explore initial program theories by other realist reviews [20].

### Literature Search

Searches were conducted on the following databases from January 2002 till December 2024: MEDLINE, CINAHL, PsycInfo, Web of Science, Scopus, Embase, and gray literature databases called Overton and Policy Commons. Hand searching of reference lists of reviews was conducted to identify any additional studies. The research team started searching from January 2002, as technologies such as smart pillboxes (devices that store and dispense scheduled medication doses at correct times, provide medication intake reminders, and monitor medication adherence by recording when the user opens the pillbox), for example, Pivotell, were first used extensively in the United Kingdom in that year [21]. Thus, these search dates increased the likelihood of inclusion of studies related to recent digital technologies designed to improve medication adherence such as smart pillboxes, automated interactive voice recognition, and mobile apps [22]. The search terms were decided based on the population (eg, multiple chronic conditions), intervention (eg, digital intervention), and outcomes (eg, medication adherence) explored in this review. The full search terms are presented in Multimedia Appendix 2.

### Screening and Selection

The papers were assessed for eligibility based on the inclusion and exclusion criteria presented in Table 2.

Titles and abstracts were screened by the first author using the web-based systematic review platform Rayyan [23] and around 10% (200/2152) of the papers were also screened by the other members of the research team for inclusion. Any disagreements were resolved through discussion.

**Table 2.** Inclusion and exclusion criteria used for selecting studies in the review.

	Inclusion	Exclusion
Population	Adults diagnosed with a chronic disease and taking long-term medicines to manage it.	Individuals receiving medication adherence support in nursing homes and in-patient settings such as hospitals and prisons, as the medications are managed by professionals in these contexts [24,25]. Individuals younger than the age of 18 years. Individuals having fluctuating capacity to consent, such as those diagnosed with dementia or substance abuse disorders. Studies that focus on one specific illness diagnosis, as the CMOs^a^ from the review were triangulated with those from the cocreation workshops, and the workshop discussions were centered around a device aimed at improving adherence for people diagnosed with any type of chronic illness. Additionally, papers focusing on only one type of chronic illness are unlikely to provide detailed information for program theory development as the information reported would be relevant to specific chronic illness diagnoses only.
Intervention	Studies reporting an intervention to improve medication adherence or reduce unintentional nonadherence with at least one digital component such as websites, mobile apps, or other digital devices. No restrictions were applied on the type of digital adherence intervention to reduce unintentional nonadherence.	Digital medication adherence interventions that only include content designed to reduce intentional nonadherence, such as education about the importance of taking prescribed medications correctly. This was based on previous studies that have consistently shown that multicomponent interventions are more effective in improving adherence as compared to interventions involving patient education only [26,27]. This criterion was also used because the workshop discussions (added qualitative data) were aimed at developing CMOs of an example device aimed at reducing unintentional nonadherence.
Outcomes	Studies assessing changes in medication adherence or unintentional nonadherence and/or contexts and mechanisms and outcomes of the interventions.	Did not report medication adherence and/or unintentional nonadherence and/or their related contexts, mechanisms, and outcomes.
Document types	Consistent with the guidelines for conducting realist reviews [14,28], no restrictions were placed on study designs. Reviews, opinion papers, and gray literature reports were included if they provided sufficient information about contexts, mechanisms, and outcomes.	Conference abstracts, paper corrections, intervention protocols, retractions, and book reviews as they are unlikely to have sufficient information for program theory development.

a CMO: context-mechanism-outcome.

### Data Extraction

A data extraction sheet was used to document details regarding authors, year of publication, study title, document type (eg, systematic review and pre-post intervention study), participants, and rigor, richness, and relevance scores. The data extraction sheet is presented in Multimedia Appendix 3 [3,9-11,29-107]. The rigor of the studies was judged based on the credibility and trustworthiness of the methodology, for example, sample size, data collection, and analysis methods [13]. The rigor of gray literature, opinion pieces, and book chapters was assessed based on significance, objectivity, coverage of the topic, authority, and accuracy [28]. Relevance was judged by assessing the extent to which papers presented information related to the topic of the review and provided evidence for program theory development. Richness was judged by assessing the extent to which papers provided details regarding mechanisms, contexts, and outcomes of the interventions [12,13]. The scoring criteria used in the realist review by Waldron et al [12] were used to assess the rigor, relevance, and richness. Studies were scored on a scale of 0 (very poor) to 3 (very good) in terms of relevance and rigor, and 0 (nothing of interest or not focused on design, implementation, or use) to 4 (much valuable data) in terms of richness. This helped in understanding the extent to which each study contributed to program theory development and the extent to which each program theory was supported by relevant and rigorous evidence [12,13].

### Data Synthesis

The lead author manually annotated the papers for program theory ideas with the help of NVIVO (Lumivero, version 14) software. Data from the literature and workshops were analyzed separately. Patterns of context, mechanisms, and outcomes from the studies included were explored by using inductive and deductive logic, and relevant text to support each program theory was documented. The recordings of the workshop discussions were transcribed verbatim. The lead author reviewed the transcripts and the participant and facilitator notes of the workshop discussions and coded the patterns of contexts, mechanisms, and outcomes. Anonymized participant quotes and participant and facilitator notes supporting each CMO were documented.

The CMOs from the realist review were then triangulated with the CMOs from the cocreation workshops by noting the extent to which the CMOs from the workshops corroborated or not with the CMOs derived from the literature. After that, a final list of program theories in different theory areas and the number of CMOs from the literature, workshops, or both were noted. After the initial CMOs were created, an iterative process of revising and refining was conducted through discussion with the research team.

### Ethical Considerations

This study received ethics approval from the Clinical Research Sponsorship ethics committee at Lancaster University and the Health Research Authority and Health and Care Research Wales ethics committee on February 4, 2025 (ethics board: social care; reference number: 24/IEC08/0037).

## Results

### Characteristics and Quality of Studies

**Figure 1.**
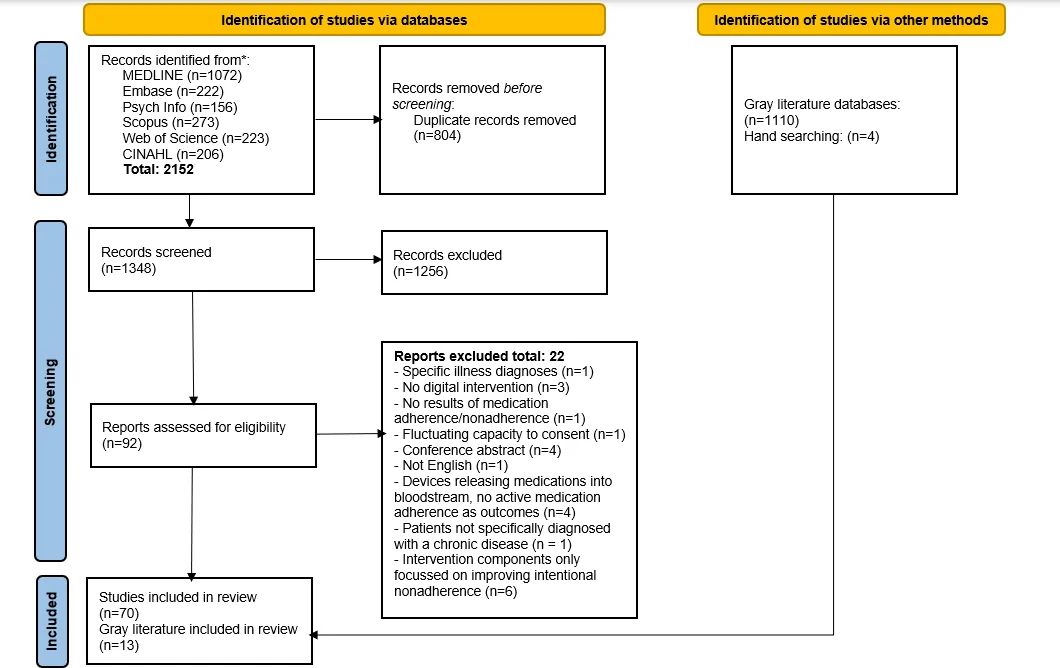
PRISMA (Preferred Reporting Items for Systematic Reviews and Meta-Analyses) flow diagram [109] with details regarding the inclusion of studies.

Eighty-three studies were included in this review. Figure 1 depicts the PRISMA (Preferred Reporting Items for Systematic Reviews and Meta-Analyses) diagram with details about the search and screening process.

Eighty-three papers, including peer-reviewed research papers (n=70) and gray literature (n=13), were included in the review. Of the research papers, 44 were narrative, systematic, or scoping reviews or meta-analyses, and 3 papers were expert opinion pieces. Of the 23 primary studies included, the largest number were conducted in the United States (n=9), with studies also conducted in Canada (n=4), Spain (n=2), the United Kingdom (n=2), China (n=2), Australia (n=1), and Switzerland (n=1). The country in which the study was conducted was not reported by 2 studies [29,30]. The primary studies included 17 intervention studies [3,10,29-44]. The rest were qualitative or quantitative studies that explored participants’ views (where participants were individuals diagnosed with chronic conditions, informal caregivers of patients diagnosed with chronic conditions, and health care professionals) regarding the components of digital medication adherence interventions [43-48].

Most of the studies were assigned a score of 3 in the “rigor” domain. However, approximately 60% (50/83) of the studies were assigned a score of 1 or 2 in the richness domain due to lack of detail regarding contexts and mechanisms related to the outcomes of the interventions. This was primarily because these studies did not report the content and delivery of the interventions in sufficient detail. Approximately 24% (20/83) of the studies were assigned a score of 1 in the “relevance” domain due to lack of data for program theory development. The authors triangulated data from studies that had low “rigor” scores with the data from studies that had higher scores while developing program theories [13,108]. The studies that had low “relevance” and “richness” scores were included in the review if they provided enough information for program theory development and if the data provided by them could be supported by the studies that were assigned high scores in these domains. The authors also transparently reported if a certain program theory was not supported adequately by rigorous, relevant, and rich data [108]. Details about the studies included in the review are presented in Multimedia Appendix 3.

Thirty-five CMOs grouped in 7 theory areas were derived from the review, cocreation workshops, or both. A narrative summary of the CMOs derived from the literature, cocreation workshops, or both in each theory area is presented in the following section. The CMOs are presented in Multimedia Appendix 4 [32,35,38,40-42,45,47,48,52,56,74,79,87,97,100,106], along with specific quotes to support them that were derived from workshop discussion recordings and/or studies in this review.

### Theory 1: Building and Maintaining Medication Intake Habits

#### Overview

**Figure 2.**
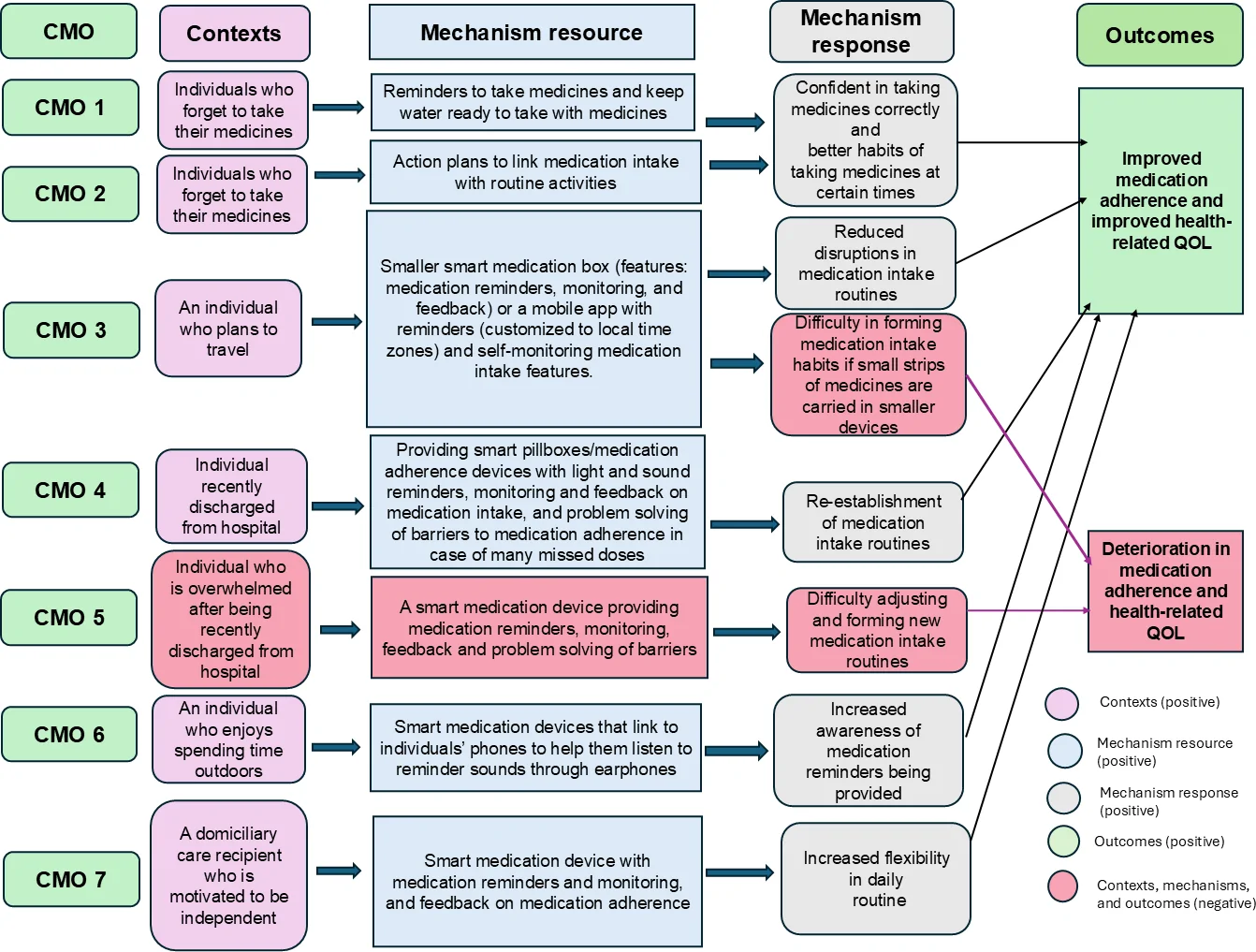
The overarching figure showing the CMOs in theory 1: building and maintaining medication intake habits. CMO: context-mechanism-outcome; QOL: quality of life.

This theory area includes the CMOs related to ways by which digital interventions facilitate the development and maintenance of effective medication intake habits. The overarching program theory based on the CMOs of theory 1 is presented in Figure 2 (see also Multimedia Appendix 4).

#### CMOs Derived From Both Literature and Workshops

Providing medication intake reminders using various devices, including mobile apps, text messages, or smart medication dispensers, can help individuals to form effective medication intake habits [10,31-46,49-97]. Additionally, workshop participants suggested that it was important to prompt individuals to keep water ready and near the smart medication device, as this can help individuals to establish habits of undertaking the necessary actions before medication intake. However, it is important to tailor the frequency and timing of reminders according to individual needs. Providing reminders at the exact times when patients plan to take their medicines was recommended to avoid excessive intrusiveness in individuals’ lives (CMO 1) [58].

Monitoring individuals’ medication adherence using smart medication devices or mobile apps and collaboratively developing action plans with them to link their medication intake with daily routine tasks, such as mealtimes [9,30,39,87,95], can also help in forming effective medication intake habits. Over time, these tasks could act as cues for medication intake, resulting in lower chances of forgetfulness and improved adherence (CMO 2).

Certain situations could disrupt existing habits related to medication intake. Hospitalization was identified as one such situation, and it could be difficult to form new and effective medication intake habits after hospital discharge, as hospitalization commonly results in medication regimen changes [35]. Thus, providing patients with smart medication devices that provide medication intake reminders and allow monitoring and feedback on medication adherence can lead to the re-establishment of effective medication intake habits and improvement in adherence (CMO 3 [35]). However, some individuals may be overwhelmed by the recent hospitalization and may not want any more changes to their routines after discharge, resulting in refusal to use smart medication devices. This could be a risk if the individual does not have any informal caregivers or care providers to help them take their medications correctly, which can potentially result in poor medication adherence and rehospitalization (CMO 4) [35].

Traveling could also lead to disruption in medication intake habits. The workshop discussions centered around a device big enough to effectively store all the prescribed medications, including liquids, but too difficult to carry while traveling. Therefore, it was suggested that the device should be left at home while traveling and individuals could use a mobile app to receive medication intake reminders and self-monitor their medication intake. Additionally, a qualitative study highlighted that mobile apps should provide medication intake reminders that are customizable to match local time zones when the individual is traveling [40]. The use of smaller smart medication devices while traveling was also suggested by a few workshop participants. However, this could lower medication adherence if an individual takes out small strips of medications to carry with them, as they would not have access to instructions regarding medication intake that are printed on medication boxes (CMO 5).

#### CMOs Derived Only From Workshops

Domiciliary care providers often visit domiciliary care recipients to prompt them to take their medications on time. Therefore, workshop participants mentioned that this device could help care recipients be more independent and flexible as they do not have to wait for care providers to visit them to take their medications (CMO 6). Workshop participants also discussed how medication reminder sounds provided by the device may not be heard by some individuals if they spend a lot of time outdoors, for example, in their garden. Thus, it was suggested that the device could be connected to individuals’ mobile phones so that they could hear the reminder sounds through earbuds while engaging in activities outdoors (CMO 7).

### Theory 2: Solving Medication-Related Barriers to Adherence

#### Overview

**Figure 3.**
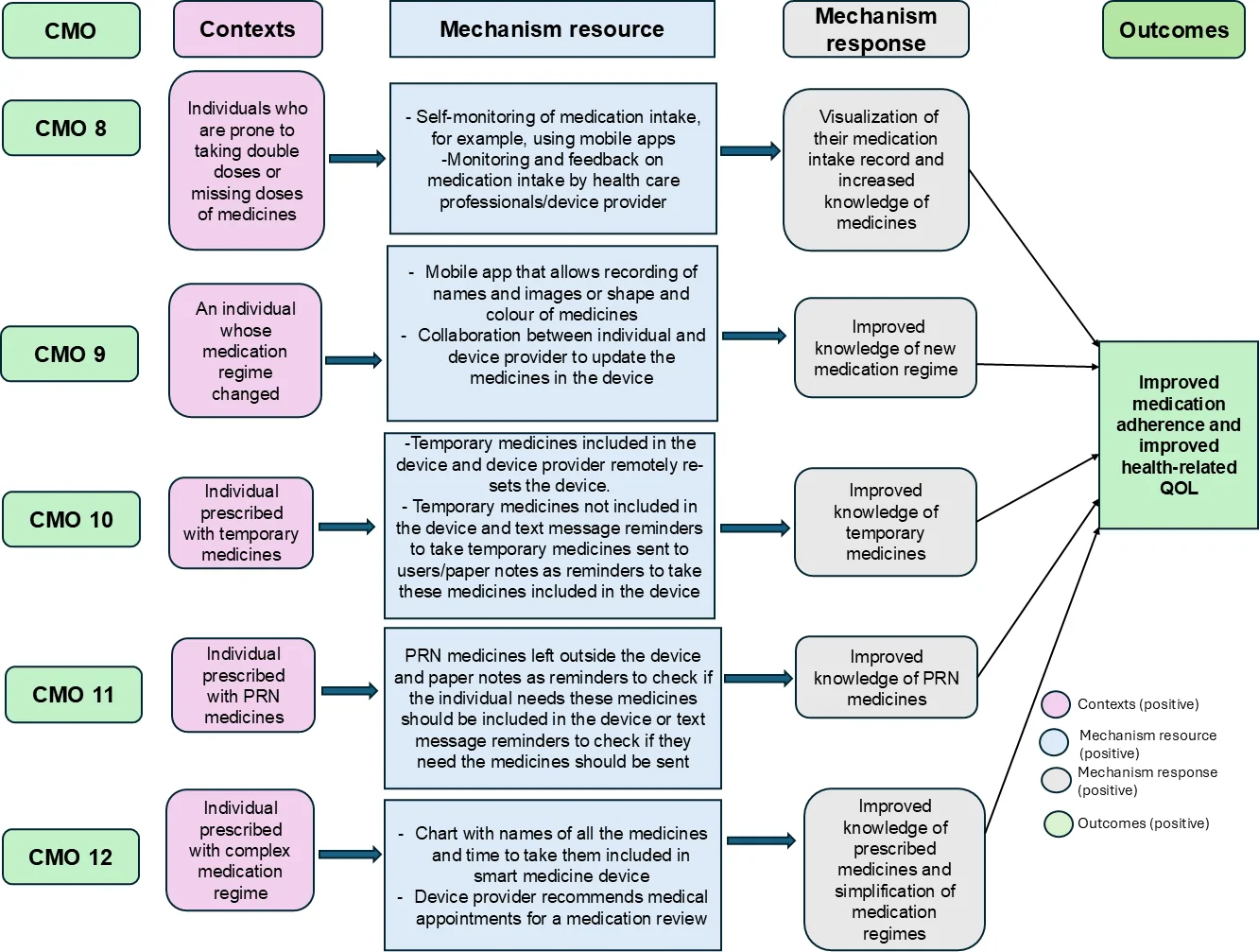
The overarching figure showing the CMOs in theory 2: solving medication-related barriers to adherence. CMO: context-mechanism-outcome; QOL: quality of life; PRN: pro re nata.

This theory area includes the CMOs related to ways by which digital interventions help in solving medication-related barriers to adherence, such as complex regimens. The overarching program theory presented in Figure 3 is based on the CMOs of theory 2 presented in Multimedia Appendix 4.

#### CMOs Derived From Both Literature and Workshops

Some individuals may be more prone to frequently missing doses of medications or taking double doses due to factors such as forgetfulness. Digital medication adherence interventions such as mobile apps can help in improving adherence by allowing individuals to self-monitor their medication intake. This can reduce chances of double doses or missed doses of medications due to individuals’ increased awareness of their own medication intake [3,11,45,46,48,52,54-56,63,73,88,96,98]. Smart medication devices, which allow a health care professional or provider of the smart medication device to monitor and provide feedback on an individual’s medication adherence, can also help in reducing chances of missed doses and double doses, as they can increase individuals’ knowledge of their medications (CMO 8) [9,29,30,39,48,52,59,63,79,85,86,89,93,96,98].

Individuals’ medication adherence can also be reduced because of medication regimen changes, as new medication intake habits may need to be established. In this context, mobile apps that enable recording of medication images or that include an option of adding the shape and color of medications along with names of the medications can help improve the recognition of new prescribed medications [32,40]. Mobile apps that announce medication names and show the pictures of the medications while providing medication intake reminders can also help in improving medication adherence by improving recognition and knowledge of the medications [40]. Additionally, workshop participants suggested that there should be collaboration between the device provider and the user of the smart medication device to update the device with the new medicines (CMO 9). This could be done effectively if the device provider regularly reminds the user to report any changes in medication regimens.

Prescription of temporary medicines such as antibiotics can also lead to reduction in medication adherence due to required changes in medication intake habits. An intervention study that provided patients with smart pillboxes before hospital discharge [35] did not include temporary medications inside the pillbox and instead provided individuals with text message reminders to take their temporary medications. Similarly, workshop participants also suggested that the temporary medications should be kept outside the smart medication devices as they could interfere with monitoring of the adherence of other medicines, and a note could be included inside the device to remind them to take their temporary medications. Alternatively, workshop participants suggested that these medicines could be kept inside the device, and the device provider could remotely reset the device after the individual no longer needs to take them (CMO 10).

Workshop participants also suggested that pro re nata (PRN) or “when required” medicines such as laxatives should be kept outside the smart medication devices as they can interfere with monitoring of other medicines. Participants also suggested that hand-written notes reminding individuals to check if they need to take the PRN medicines should be included in the device (CMO 11). Similarly, the intervention study by Shahani et al [35] that provided patients recently discharged from hospital with smart pillboxes also did not include PRN medications inside the pillboxes. Individuals were instead sent text message reminders to check if they needed to take these medications.

#### CMO Derived Only From Workshops

Workshop participants acknowledged that complex medication regimens could make medication adherence challenging. The smart medication device that they were discussing provided users with reminders to take medications but did not specify the name of the medication and the time to take it. Thus, participants suggested that a written chart of the names of medications and the correct time to take each medicine should be included in the box. This can improve individuals’ knowledge of medication regimens and medication adherence. Additionally, the device providers mentioned that they would recommend the individual schedule an appointment with the pharmacist or general practitioner for a potential medication review to simplify medication regimes (CMO 12).

### Theory 3: Enabling Collection of Prescription Medications on Time

**Overview**

**Figure 4.**
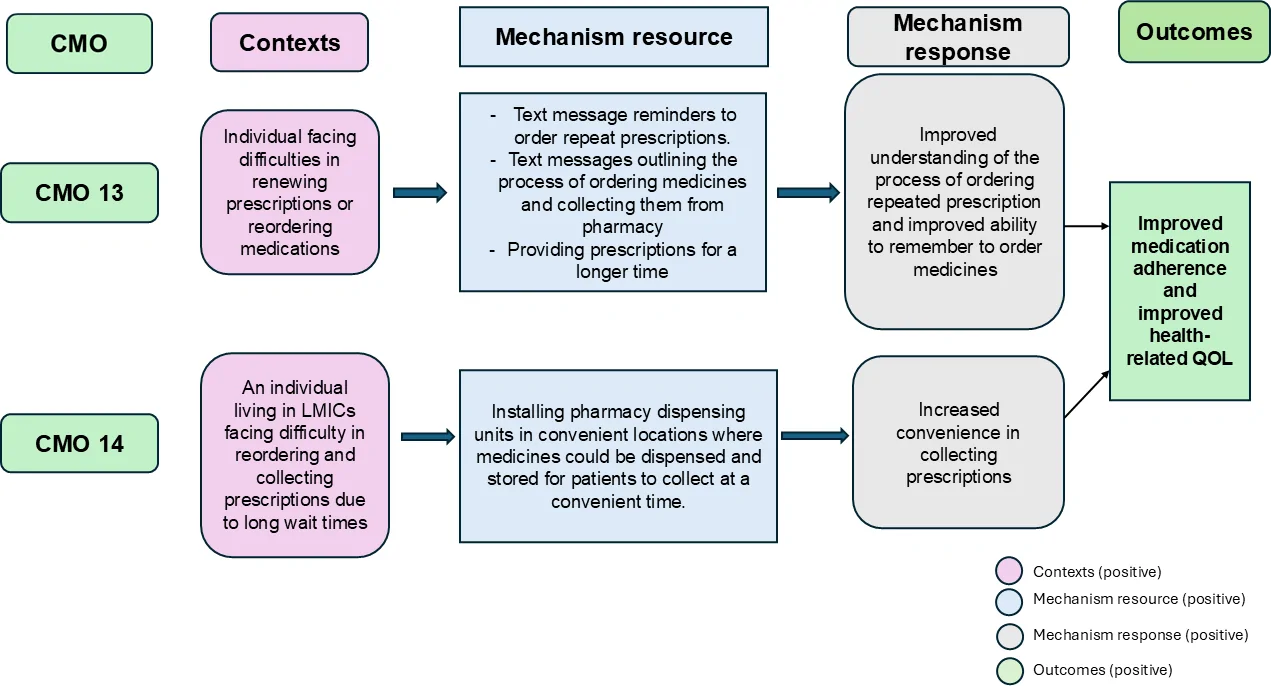
The overarching figure showing the CMOs in theory 3: enabling collection of prescription medications on time. CMO: context-mechanism-outcome; LMIC: low- and middle-income country; QOL: quality of life.

This theory area includes the CMOs related to ways by which digital interventions can improve collection of prescription medications on time. These CMOs were derived from the literature only. The overarching program theory presented in Figure 4 is based on the CMOs of theory 3 presented in Multimedia Appendix 4.

Studies have highlighted the importance of enabling easier access to prescription medications to prevent primary nonadherence. Text message reminders or reminders from mobile apps to order prescription medications on time and outlining the exact process of ordering prescriptions and collecting them can improve medication adherence. This would be helpful for individuals who forget to order prescriptions or who are unable to understand the process of ordering repeated prescriptions. Additionally, in the context in which frequent medication reviews are not required, providing repeat prescriptions for longer periods of time can improve medication adherence as it would reduce the frequency of ordering prescriptions (CMO 13) [48,68,79,80,99].

Mash et al [100] suggested that collecting prescription medications on time could be particularly challenging for individuals living in low- and middle-income countries (LMICs) due to limited human resources and poor infrastructure in primary health care services, resulting in long waiting periods to collect prescriptions. Thus, this study [100] suggested the installation of pharmacy dispensing units which allow remote dispensing of medications (medication dispensing can be requested online by patients) and storage of medicines in password-protected lockers in convenient areas. This can allow patients to collect their medicines at a convenient time and help in improving medication adherence (CMO 14). However, installation of pharmacy dispensing units requires high start-up costs. Additionally, the long-term efficacy, feasibility, and acceptability of these technologies are yet to be determined, as these have been piloted on a very small scale only in South Africa [77,100].

### Theory 4: Supporting People With Sensory or Motor Impairments and Unpredictable Health Issues

#### Overview

**Figure 5.**
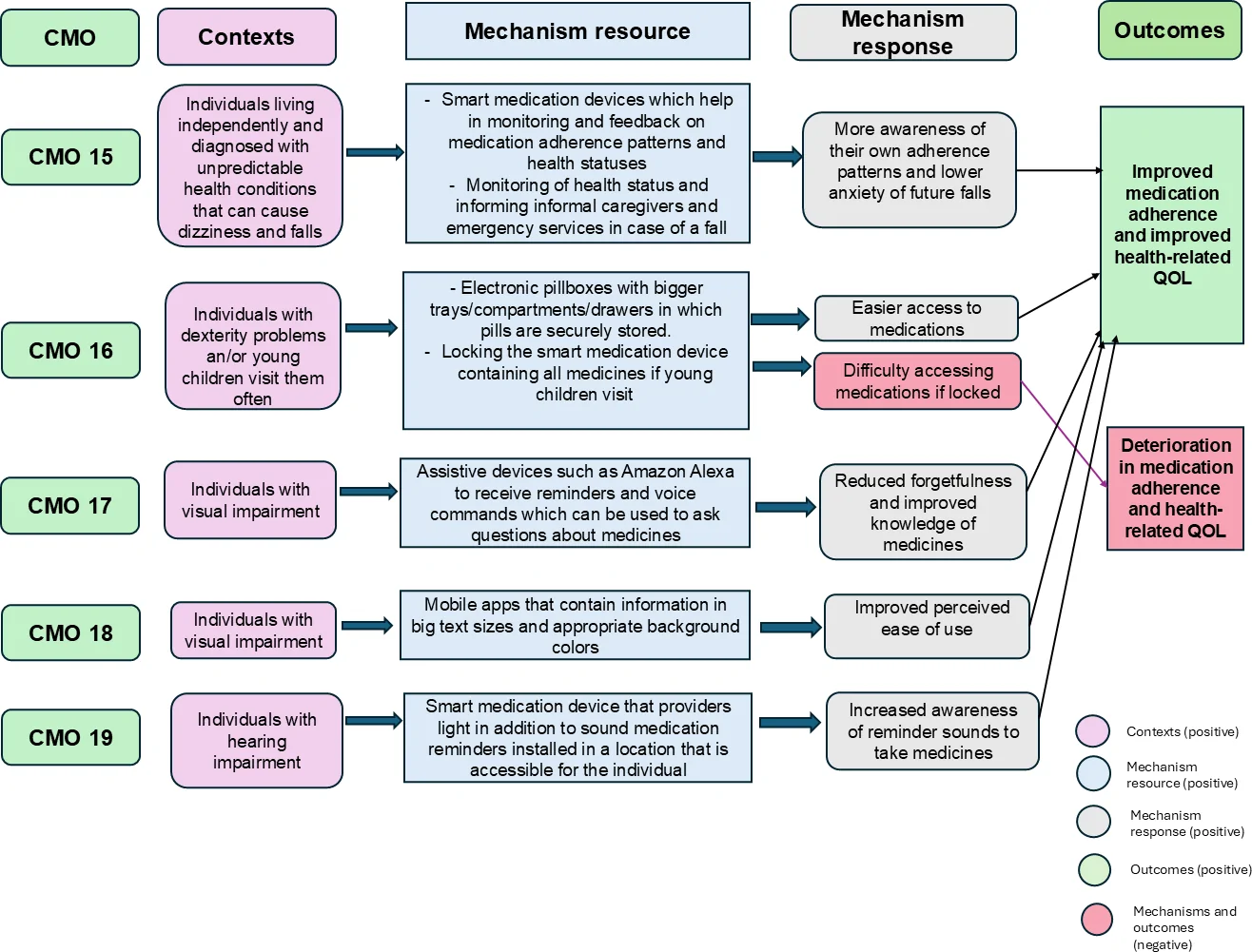
The overarching figure showing the CMOs in theory 4: supporting people with sensory or motor impairments and unpredictable health issues. CMO: context-mechanism-outcome; QOL: quality of life.

This theory area includes the CMOs related to ways by which digital medication adherence interventions can improve the adherence of people with unpredictable health conditions and sensory and motor deficits. The overarching program theory presented in Figure 5 is based on the CMOs of theory 4 presented in Multimedia Appendix 4.

#### CMOs Derived From Both Literature and Workshops

Smart medication devices that allow health care professionals and device providers to monitor medication adherence and provide feedback on adherence patterns based on the monitoring can improve individuals’ adherence by increasing their awareness of their adherence patterns. Changes in the health status of individuals could be detected by observing any changes in the frequency of individuals’ use of devices (CMO 15). If an individual misses several doses of medications, a timely intervention could be provided [30,33-35,42,52,56,57,60,61,64,65,73,78,81-83,86,88,95,99,101-103]. Emergency services and informal caregivers could be contacted in the context of severe deterioration of health or a fall. Thus, these devices can also be used for effective telecare monitoring in situations where older adults live alone [52].

However, some individuals with dexterity issues, for example, people with arthritis, may struggle to use devices such as smart pillboxes, as it may be difficult for them to take the medicines out of the containers. Providing these individuals with smart pillboxes with bigger compartments or pill trays could enable easier access to medications [34,42,56,73,74]. Additionally, workshop participants highlighted that if an individual is visited often by young children, the smart medication devices might need to be locked or kept out of reach of children. However, if an individual has poor dexterity and struggles to open locks, they might struggle to unlock the devices to access their medications, resulting in poor medication adherence (CMO 16).

#### CMOs Derived Only From the Literature

Several studies recommended that the mobile apps aiming to support individuals’ medication adherence should include bigger text sizes and appropriate backgrounds to enable individuals with visual impairments to see the content properly (CMO 17) [40,91]. Devices such as Amazon Alexa that provide auditory medication intake reminders can also be helpful in improving medication adherence among individuals with visual impairment (CMO 18). These devices also have voice command features which can be used to ask questions regarding the prescribed medications, which can enable a better understanding of the prescribed medicines, thereby improving medication adherence [41,74].

#### CMO Derived Only From Workshops

As described earlier (Methods section), the smart medication device discussed in the workshops provided light and sound medication intake reminders. Thus, participants felt that the device was suitable for individuals with visual and hearing impairments. However, participants mentioned that individuals with hearing impairment may not be able to hear the reminder sounds if it is not installed in an accessible location. Thus, installing the device at an accessible location in individuals’ homes was identified as a useful mechanism to improve the adherence of individuals with hearing impairment (CMO 19).

### Theory 5: Supporting Individuals With High Anxiety, Low Medication Self-Efficacy, and Low Social Support

**Figure 6.**
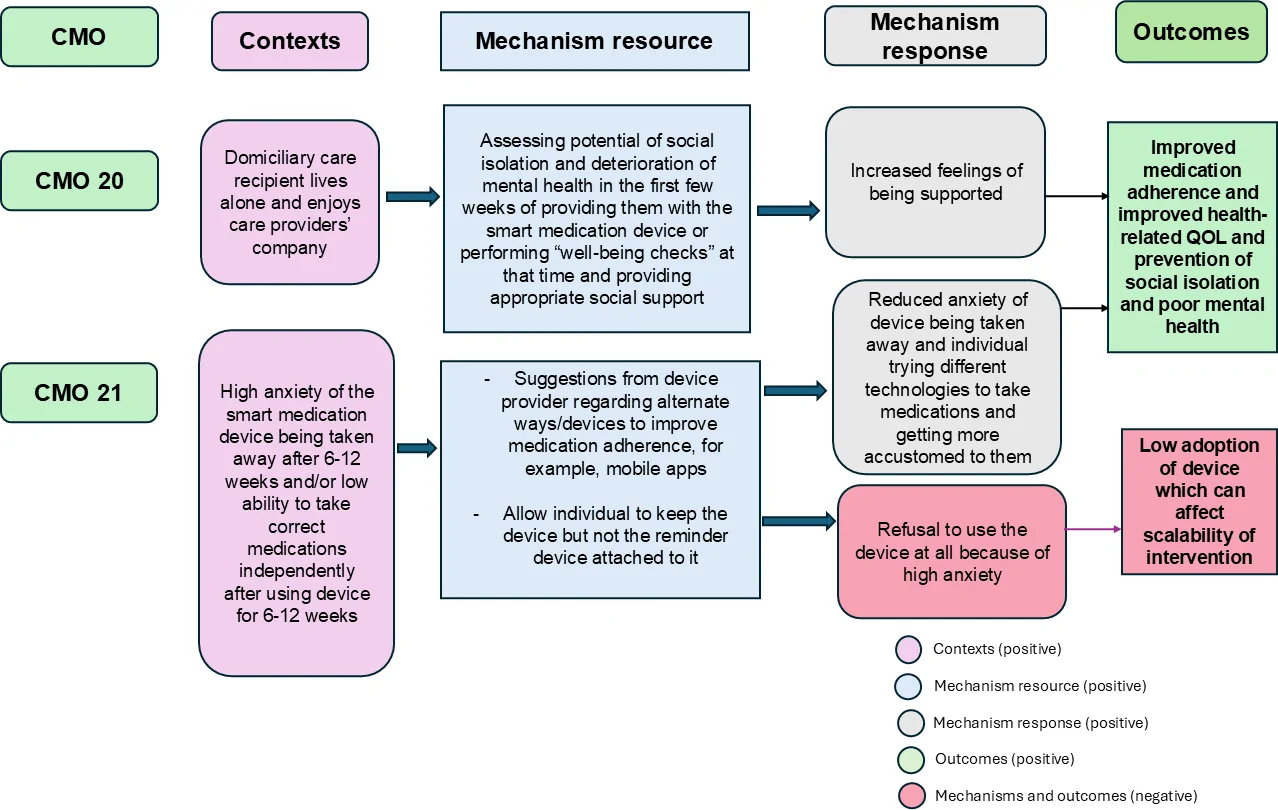
The overarching figure showing the CMOs in theory 5: supporting individuals with high anxiety, low medication self-efficacy, and low social support. CMO: context-mechanism-outcome; QOL: quality of life.

This theory area includes the CMOs related to how the smart medication device that was provided to domiciliary care recipients and hospital discharge patients in the United Kingdom could help in improving the medication adherence of patients with high anxiety, low self-efficacy, and low social support. The CMOs in this section were derived from the workshop discussions only. The overarching program theory presented in Figure 6 is based on the CMOs of theory 5 presented in Multimedia Appendix 4.

Domiciliary care recipients in the United Kingdom who have difficulties with medication adherence are generally visited by care providers to prompt them to take their medications. Therefore, participants discussed that if a domiciliary care recipient lives alone and has low social support, they may enjoy the company of care providers and look forward to their visits. These care recipients may feel isolated if the care providers’ visits are reduced when they self-manage their medicines. Thus, participants suggested that the device provider should assess the mental health and the potential for social isolation of these individuals and provide appropriate social support after the device is installed in their homes. This is especially important for individuals with a prior history of mental health diagnoses such as depression (CMO 20).

As mentioned in the Methods section, this device was provided to the recipients for only 6‐12 weeks, and during that time, the device was aimed at helping them to develop effective medication intake habits, after which the device was taken away. Participants mentioned that the removal of the device could cause anxiety among some individuals as they may become dependent on it for maintaining their medication adherence. Participants recommended that users could be allowed to keep the device that stores all the medicines safely, but the electronic reminder, which is used to provide medication intake reminders and monitor individuals’ adherence (the costly part of the device), could be taken away by the device provider. This could ensure that all the medicines are stored in the same place and strengthen individuals’ habits of taking medications from the same place, resulting in reduced anxiety and maintenance of medication adherence. However, some individuals with high anxiety may refuse to use the device at the outset if they are informed that it would be taken away after 6‐12 weeks. This could reduce the large-scale adoption and scalability of the intervention. The device provider also mentioned that they could suggest, following removal, alternative devices to maintain their medication adherence, for example, mobile apps. This could help individuals to try different types of technology to manage their medications and improve their comfort with technology (CMO 21).

### Theory 6: Supporting Individuals Who Are Uncomfortable With Technology or Certain Aspects of Technology

#### Overview

**Figure 7.**
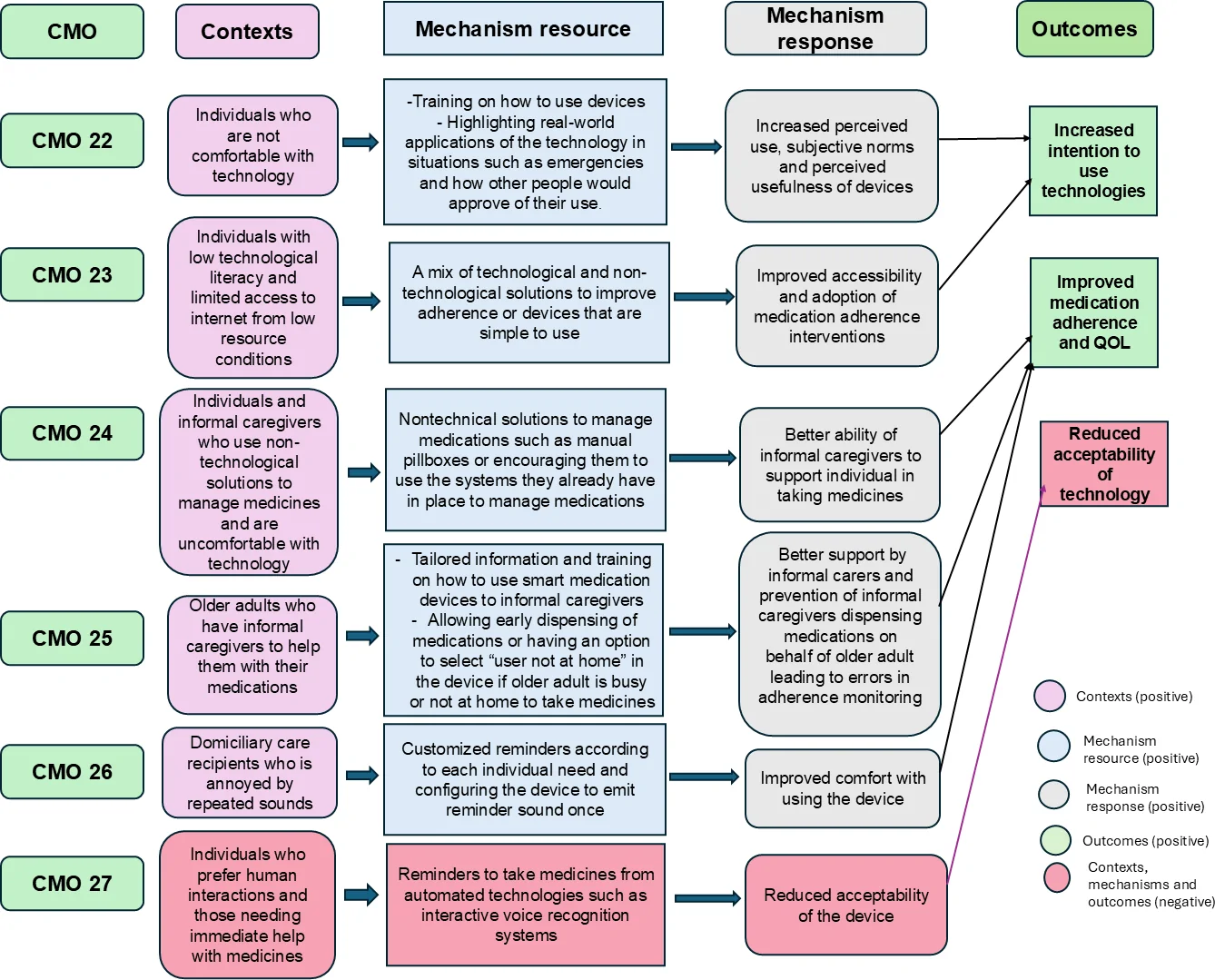
The overarching figure showing the CMOs in theory 6: supporting individuals who are uncomfortable with technology or certain aspects of technology. CMO: context-mechanism-outcome; QOL: quality of life.

This theory area includes CMOs related to mechanisms by which digital medication adherence interventions can improve the adherence of individuals who are uncomfortable with technology or certain aspects of technology, such as repeated reminder sounds for taking medications. The overarching program theory presented in Figure 7 is based on the CMOs of theory 6 presented in Multimedia Appendix 4.

#### CMOs Derived From Both Literature and Workshops

Workshop participants mentioned that it was important to provide some training on how to use the smart medication device, especially to individuals who were uncomfortable with technology (CMO 22). This was corroborated by the results of a qualitative study [40] which showed that providing hands-on training on the use of a mobile app intervention in small groups could increase the perceived ease of use of the technology. Additionally, highlighting the usefulness of the mobile app in real-world situations such as health emergencies can increase perceived usefulness. Highlighting how their family members or doctors may approve of their use of the app to improve their adherence could improve subjective norm (individuals’ perception of how others would perceive their behavior). According to the Technology Acceptance Model, an increase in perceived usefulness, perceived ease of use, and subjective norms can increase the intention to use the technology to improve medication adherence [40].

Workshop participants also highlighted the importance of providing some training on how to use smart medication devices to informal caregivers or family members so that they can better support the person they care for. Additionally, some of the smart medication dispensers that provide medication intake reminders, allow monitoring of adherence, and dispense medications according to individual doses may need to be adapted for use if informal caregivers support individuals with their medication intake [42]. Some of these devices assume that the medication has been taken by the individual correctly once the device has been opened and medications are dispensed [42]. However, if the individual is busy or not at home, informal caregivers may dispense the medication on behalf of the individual. Therefore, these devices should have an option to select “user not at home” to indicate that the individual is not at home or is busy [42] to improve the accuracy of medication adherence monitoring (CMO 23).

Some studies suggested that nontechnological solutions or a combination of technological and nontechnological solutions may be more suitable to improve the adherence of individuals living in low-resource conditions and LMICs. This is important as poor infrastructure, low internet access, and technological literacy among individuals are common in these contexts [46,77,104,105]. This was corroborated by workshop discussions as participants noted that one of the advantages of the smart medication device discussed was that it had a built-in SIM card and no Wi-Fi was needed to use it, which made it more accessible for individuals living in low-resource conditions (CMO 24).

#### CMOs Derived Only From the Literature

A qualitative study conducted with informal caregivers of older adults [47] showed that many informal caregivers already had established nontechnical methods of supporting medication adherence of the person they were caring for (eg, paper medication lists with descriptions of medicines and information about when to take medicines and a box to store all the medicines). Thus, the informal caregivers were unwilling to learn and use technologies such as mobile apps to support medication adherence as they felt that use of these technologies would increase caregiver burden. In these contexts, encouraging individuals to use these nontechnological solutions may be more suitable (CMO 25).

CMO 26 highlighted the low acceptability of automated technologies such as interactive voice recognition systems and automated telephone communication systems to receive medication intake reminders [38,74,85,106] among patients who preferred speaking to a person. These technologies are also unsuitable for patients needing immediate support regarding their health condition or medication intake, and using these technologies can result in lower medication adherence for these individuals [38,106].

#### CMO Derived Only From Workshops

A few care recipients who participated in the workshops mentioned that repeated medication intake reminder sounds from the device may be annoying, and this could negatively affect medication adherence of individuals if they open the device and take the wrong medications to stop the reminder sounds. Thus, participants suggested that to increase individuals’ comfort with using the technology, the reminder sounds should be customized to individual needs, and the frequency of the reminders should be reduced to only once if required (CMO 27).

### Theory 7: Economic, Policy, and Organizational Factors Affecting Implementation of Digital Medication Interventions

#### Overview

**Figure 8.**
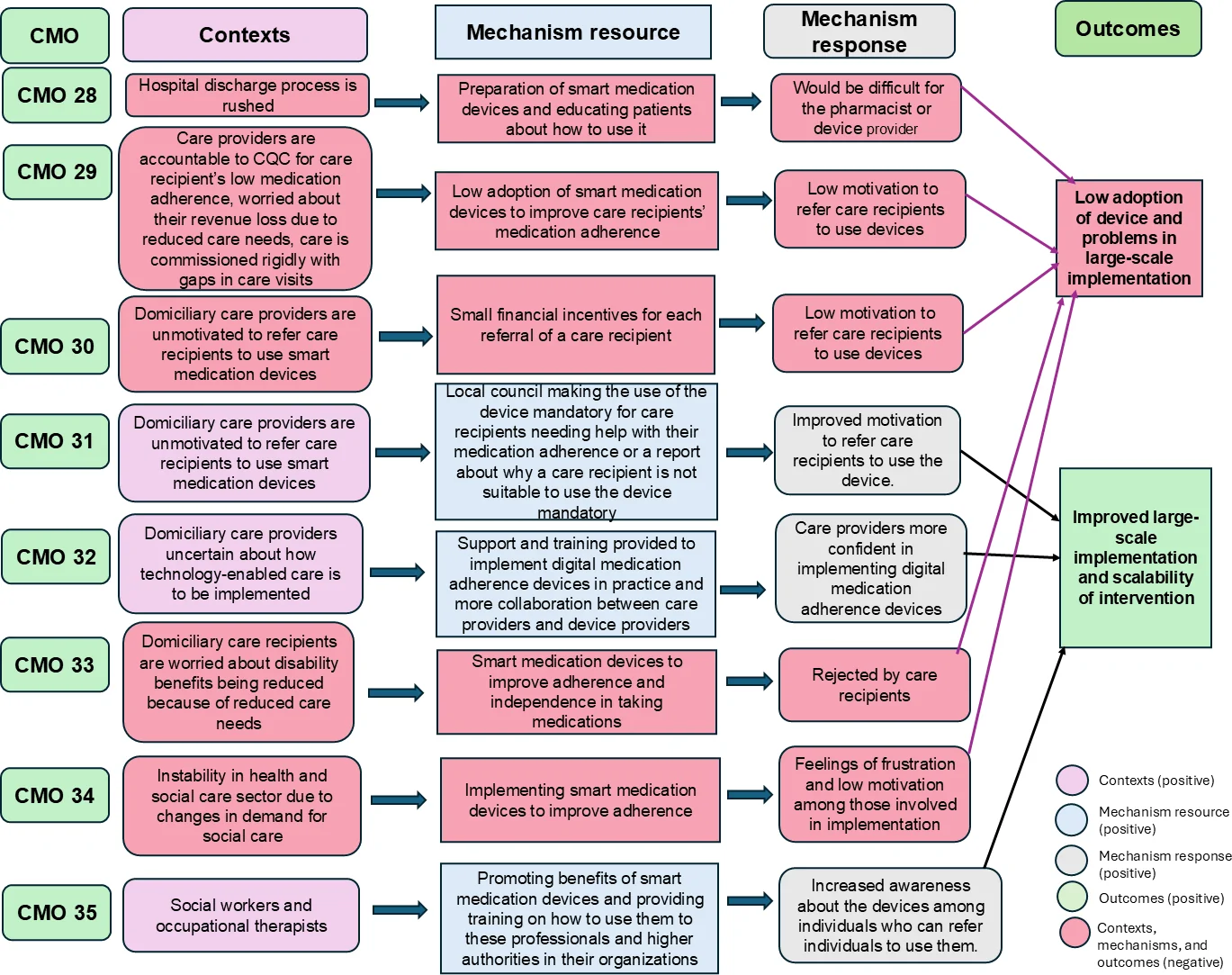
The overarching figure showing the CMOs in theory 7: economic, policy, and organizational factors affecting implementation of digital medication interventions. CMO: context-mechanism-outcome; CQC: Care Quality Commission; NHS: National Health Service.

This theory area includes CMOs related to the wider economic, policy, or workplace organizational factors that affect large-scale implementation of digital medication adherence interventions. The overarching program theory presented in Figure 8 is based on the CMOs of theory 7 presented in Multimedia Appendix 4.

#### CMO Derived Only From the Literature

An intervention study which provided patients with smart pillboxes before hospital discharge [35] highlighted that there were several barriers at the organizational level relating to implementation of the intervention. The rushed nature of hospital discharge made it difficult for pharmacists to prepare the pillbox and demonstrate the use of the pillbox before patients were discharged. Thus, the authors recommended that organizational changes such as early communication between prescribing clinicians and pharmacists preparing the pillboxes and early provision of the discharge medications are required for successful implementation in the context of hospital discharge (CMO 28) [35].

#### CMO Derived Only From Workshops

The workshop participants who were involved in implementing the device highlighted several barriers to the large-scale implementation of the device. The number of individuals who were being referred to use the device from domiciliary care provider organizations and short-term re-enablement services that provide care to patients recently discharged from hospitals in the United Kingdom was very low [110].

Participants felt that the scalability of the intervention was potentially reduced because obtaining referrals from domiciliary care organizations was particularly difficult because care providers were anxious about potential revenue loss for them. Their anxiety was further increased because of a recent increase in the number of care providers locally as compared to the demand for their services (CMO 29). Additionally, care providers have to undertake more administrative tasks to identify the care recipients who were suitable to use the device and refer them to the team implementing the device. Care providers would also have to report any lapses in medication adherence among care recipients to the Care Quality Commission that regulates the domiciliary care services in the United Kingdom [111]. Care providers were worried that they may be held accountable for any lapses in medication adherence of care recipients. Care provision is generally commissioned in the United Kingdom with rigid specifications of tasks and times of visits, with several gaps between the care visits. Early morning and late-night visits are common. Care providers prefer to undertake some medication prompting visits during the rest of the day to increase the hours of their work shift and their financial compensation.

The device provider mentioned that they offered care organizations small financial incentives (£25‐£30; a currency exchange rate of £1=US $1.35 was applicable) for each referral. However, this did not improve care providers’ motivation to refer care recipients to use the device because of fear of long-term revenue loss. A higher value of financial incentives would have also limited the cost savings that could have been achieved from this intervention (CMO 30).

Thus, participants felt that the local councils that commission care services should make the use of the device a part of usual care for care recipients needing support with their medication adherence. If an individual is not suitable for using the device, a report from care providers outlining the reasons for that should be required. Participants felt that this may have improved the implementation of the intervention (CMO 31). Additionally, the local councils were aiming to increase technology-enabled care provision [111], but care providers were uncertain about how to implement it in their care provision. Thus, participants felt that local councils should support care providers to implement technologies such as smart medication devices in their care provision and encourage more collaborations between them and care providers (CMO 32).

Participants also highlighted that care recipients may not be motivated to use the device because they could be anxious about the potential reduction of their disability benefits if they require less support for daily living tasks and mobility due to a reduction in the care providers’ visits (CMO 33) [112].

Overall, participants felt that the instability in the health and social care sector in the United Kingdom in recent years contributed to the problems with implementation. The number of care providers available and demand for their services fluctuated significantly in the previous years due to the national government’s efforts to improve their recruitment and retention. This resulted in a reduced demand for their services in the local area when the project was delivered [111,113]. Thus, participants suggested that future projects should allocate enough time at the beginning to assess contextual factors and to form effective partnerships with organizations that can help improve the implementation of devices (CMO 34). Participants also suggested that the benefits of this device should be promoted to professionals such as social workers and occupational therapists as they regularly assess the care needs of individuals. It was also important to promote the device among the higher authorities of the organizations in which these professionals work to get leadership support for the implementation (CMO 35).

## Discussion

### Summary of Findings

Thirty-five CMOs in 7 theory areas were identified. CMOs within theory 1 demonstrated ways in which devices can help establish and maintain medication intake routines, such as by providing medication intake reminders. Theory 2 presented CMOs related to solving medication-related barriers to adherence, for example, self-monitoring of medication adherence to reduce double or missed doses. Theory 3 presented CMOs related to enabling collection of prescription medications on time, for example, by sending text message reminders to collect prescriptions on time. Theory 4 included CMOs related to improving the medication adherence of individuals with sensory and motor impairments and unpredictable health conditions, for example, encouraging individuals with visual impairments to use assistive devices with voice command features such as Alexa to receive medication intake reminders. Theory 5 presented CMOs related to customizing smart medication devices according to the needs of individuals with high anxiety and low social support and low medication self-efficacy, such as assessing the mental health of individuals who are socially isolated and providing them with appropriate social support. Theory 6 presented CMOs related to supporting individuals who are uncomfortable with technology, for example, providing training on how to use smart medication devices. Theory 7 presented CMOs related to policy and economic factors that were barriers to the large-scale implementation of smart medication devices, such as a lack of suitable incentives for care providers to refer domiciliary care recipients to use the device.

CMOs were derived from both the literature and workshops (14/35, 40%), with a proportion derived only from the literature (7/35, 20%) and a proportion only from the workshops (14/35, 40%). CMOs derived from workshops provided important information about implementing smart medication devices in the United Kingdom and in the context of domiciliary care and hospital discharge. CMO 14 (enabling easier access to prescriptions in LMICs) was not adequately supported by high-quality evidence, and further research is needed to support it. After assessing the “rigor” at a program theory level, the authors excluded a CMO that was initially developed regarding ingestible digital pills for monitoring and providing feedback on medication adherence because these have recently been discontinued due to low acceptability and adoption [114].

### Comparison With Prior Work and Implications for Practice and Policy

The results of the current realist review are consistent with those of a qualitative study [114] in which patients diagnosed with cardiovascular diseases acknowledged that medication intake reminders and reminders for keeping water ready to take with medications helped in maintaining effective medication intake habits. Similarly, the importance of tailoring the timing and frequency of the reminders according to individual preferences to prevent “alert fatigue” and annoyance related to receiving repeated reminders was also highlighted [115]. The importance of action planning related to medication intake by linking it with daily routine tasks such as mealtimes [116,117] was also supported by previous studies.

Previous studies have also found that self-monitoring and monitoring and feedback on adherence by health care providers could help in improving adherence, including reducing the possibility of missed doses [118,119]. Smart pillboxes or medication dispensers that allow monitoring of adherence have also been recommended by previous studies as an effective device for telecare monitoring of older adults and individuals diagnosed with unpredictable health conditions living independently. However, these devices should also be tailored for people with visual, hearing and dexterity problems [120]. A related issue shown in a previous qualitative study highlighted the importance of locking smart medication devices to prevent any interference by children [121], but it should still be easy to open for people with dexterity problems. However, all digital medication interventions should address privacy concerns [42,45,49,59,88,103,104]. Terms and conditions educating users on data protection and ways to protect their privacy, password protection of data, and an option to consent to the collection and use of their personal data should be included [64,108].

There was only 1 study included in the review that reported factors influencing large-scale implementation and scalability of a digital medication adherence intervention [35]. However, the workshop discussions identified several economic and policy factors that significantly hampered the large-scale implementation of the smart medication device that was provided to the patients at hospital discharge and domiciliary care recipients in the ICB region, United Kingdom. Examples of this included a recent decline in demand for adult domiciliary care services resulting in care providers’ fears of revenue loss, care providers’ fears of being held responsible for medication errors by care recipients [111], and care recipients’ fears of losing disability benefits due to reduction in their care [112]. As a higher value of financial incentives would have also limited the cost savings from this intervention, policy changes to make the use of the device the usual care for individuals requiring medication prompting visits from care providers should be considered right from the beginning of any person’s care plan.

### Directions for Future Research

Only 1 study [35] included in this review focused on re-establishing effective medication intake habits after hospital discharge; this was also the only study included in the review that assessed and reported factors affecting the large-scale implementation of the intervention. While the program theories derived from the workshop discussions filled this gap to an extent, future digital medication adherence intervention studies should explore and report the wider social, policy, and economic contextual factors that affect the scalability of the interventions. This is important as insights into factors affecting scalability of previous interventions can help in developing more effective implementation strategies for future interventions [122]. Further research is also needed to explore health system and policy changes needed to effectively implement medication adherence devices in the context of hospital discharge.

Lessons learned from the cocreation workshops were that it was essential for future studies to assess the wider social, economic, and policy factors that can potentially affect the scalability before starting the implementation of an intervention. It was also important to allocate enough time at the beginning of projects to develop effective relationships with key professionals in the community who can support the effective implementation of smart medication devices. Effective implementation strategies could be co-designed with key professionals who could support large-scale implementation of digital medication adherence interventions in the community at the beginning of the project [123].

Additionally, assessment of “richness” of the studies showed that details regarding the content and delivery methods of medication adherence interventions were lacking in some studies, which somewhat hindered the exploration of additional CMOs [9,29,30,35,43,44,49,53,56,58,62,69-72,75-77,80-84,86,88,92-94,96-98,100,103-106,108]. Thus, it is recommended that future medication adherence intervention studies describe the context, content, and delivery of the interventions in detail [123]. Moreover, the wide variation in the methods used in assessing adherence made it difficult for reviews to compare results among studies [83,106]. Some studies assessed adherence using only self-report measures, which are prone to bias and overestimation of adherence. There are drawbacks in using objective methods as well; for example, many digital tools can only assess when the packaging is opened and closed but do not measure if the medication has been ingested. Therefore, assessing adherence by using a combination of objective and self-report measures has been recommended for a comprehensive assessment of adherence [124,125].

### Strengths and Limitations

A key strength of this study was its rigorous adherence to Medical Research Council guidelines [123] to uncover the contexts and mechanisms related to the efficacy or inefficacy of digital medication adherence interventions using realist synthesis. Additionally, cocreation workshops with diverse stakeholders of a project aiming to implement a digital medication adherence intervention led to the identification of CMOs of the intervention, which led to triangulation of the data. It also led to a better understanding of the implementation of such interventions in the community in the United Kingdom. Given the rapidly changing social policy and social care environment, a further strength was the use of the repeated workshops at approximately a 3-month interval with the same participants, which also gained insights as the implementation of the device evolved.

However, a limitation is that the CMOs developed from the workshop discussions could not be empirically verified and further refined using the cost-effectiveness and the effectiveness data of the device in improving medication adherence and quality of life because of the challenges in recruiting participants to use it. Nevertheless, one of the strengths of this study was that it highlighted that, in the future, when designing potential projects for interventions of this nature, it is important to assess the wider contexts and design some kind of flexibility into any project plans so that if wider contextual changes emerge, there is scope for the project to change as well.

This study focused on individuals who had adequate capacity to self-manage medication intake, and so papers which focused on medication adherence of people with severe mental illnesses, dementia, and substance use were excluded. Thus, the program theories derived from this review may not be applicable for medication adherence interventions for these populations. Only program theories associated with improving medication adherence among unintentionally nonadherent individuals were included; therefore, these program theories may not be generalizable for interventions designed for individuals who are intentionally nonadherent.

### Conclusions

Digital interventions are commonly used to improve medication adherence; however, their efficacy may be limited because of a lack of effective tailoring for different populations and contexts [8]. Thus, this realist review addresses this gap in the literature by providing recommendations for tailoring digital medication adherence interventions effectively. Medication intake reminders and reminders for keeping water ready before medication intake, self-monitoring, and monitoring and feedback on medication adherence by device providers or health care professionals were the main active ingredients of interventions to reduce unintentional nonadherence across all theory areas. That said, intervention developers and implementers should adapt these active ingredients for specific contexts and patient subgroups by considering the recommendations provided in the CMO configurations identified in this study. This is important as the same active ingredients will not be effective across contexts because of how contextual factors or patients’ responses interact with the mechanism resources. For example, a person with visual impairment may be better supported by a device that provides auditory medication intake reminders (eg, Alexa) rather than pillboxes or mobile apps. Some CMOs in this paper suggest different delivery methods of medication adherence interventions in specific contexts; for example, CMO 11 suggests not including PRN medicines in medication devices and instead providing text message reminders or including written notes in the device as reminders to take them. In these situations, the intervention implementers should choose the delivery methods that align with the patients’ needs and preferences [123] and avoid implementing the mechanisms that can potentially result in unintended or negative consequences.

The review also provided insights into the economic, policy, and organizational factors affecting implementation and ways to overcome them. However, further studies exploring these factors are required, especially in the context of domiciliary care provision and hospital discharge. Future interventions should report intervention content and delivery in detail and codevelop effective implementation strategies with professionals who can support the large-scale implementation [123].

## Supplementary material

10.2196/89803 Multimedia Appendix 1 Cocreation workshop details.

10.2196/89803 Multimedia Appendix 2 Realist review search strategies.

10.2196/89803 Multimedia Appendix 3 Data extraction sheet.

10.2196/89803 Multimedia Appendix 4 Context-mechanism-outcomes and quotes supporting the context-mechanism-outcomes.

## Abbreviations

CMO: context-mechanism-outcome
ICB: integrated care board
LMIC: low- and middle-income country
PRISMA: Preferred Reporting Items for Systematic Reviews and Meta-Analyses
PRN: pro re nata
PROSPERO: The International Prospective Register of Systematic Reviews
RAMESES: Realist and Meta-Narrative Evidence Synthesis: Evolving Standards
