# Supplementary material for: Contexts and Mechanisms Related to the Efficacy of Digital Medication Adherence Interventions to Support Medication Adherence Among Adults With Chronic Diseases: Realist Review
Source: Interact J Med Res. 2026 Aug 28;15:e89803. doi: 10.2196/89803 (PMC13524365; doi:10.2196/89803)
Supplement: Supplementary file 2 [file ijmr-v15-e89803-s002.docx]

**Search strategies**

**Psych info, Medline, CINAHL**

(MH "Medication Adherence+") OR TI ( ((medication* OR drug* OR pharma*) N3 (adheren* OR complia* OR non-adheren* OR "non adheren*")) ) OR AB ( ((medication* OR drug* OR pharma*) N3 (adheren* OR complia* OR non-adheren* OR "non adheren*")) )

# AND

( (MH "Chronic Disease+") OR (MH "Multiple Chronic Conditions") OR (MH "Long-Term Care") ) OR AB ( (("long term" or "chronic" or "longlasting or "persistant") N3 (condition* or "disease" or "illness" or "sickness")) ) OR TI ( (("long term" or "chronic" or "longlasting or "persistant") N3 (condition* or "disease" or "illness" or "sickness")) )

# AND

(( MH "digital intervention" or technology or internet or "mobile application") ) OR ( ( ((digital or "mobile app*" or technolog* or "internet") N3 (intervention* or therap* or strateg*)) ) ) OR ( ( ((digital or "mobile app*" or technolog* or "internet") N3 (intervention* or therap* or strateg*)) ) )

**EMBASE (Ovid)**

(MH "Medication Adherence+") OR TI ( ((medication* OR drug* OR pharma*) adj3 (adheren* OR complia* OR non-adheren* OR "non adheren*")) ) OR AB ( ((medication* OR drug* OR pharma*) adj3 (adheren* OR complia* OR non-adheren* OR "non adheren*")) )

# AND

( (MH "Chronic Disease+") OR (MH "Multiple Chronic Conditions") OR (MH "Long-Term Care") ) OR AB ( (("long term" or "chronic" or "longlasting or "persistant") adj3 (condition* or "disease" or "illness" or "sickness")) ) OR TI ( (("long term" or "chronic" or "longlasting or "persistant") adj3 (condition* or "disease" or "illness" or "sickness")) )

(( MH "digital intervention" or technology or internet or "mobile application") ) OR ( ( ((digital or "mobile app*" or technolog* or "internet") adj3 (intervention* or therap* or strateg*)) ) ) OR ( ( ((digital or "mobile app*" or technolog* or "internet") adj3 (intervention* or therap* or strateg*)) ) )

**Web of Science**

(MH "Medication Adherence+") OR TI ( ((medication* OR drug* OR pharma*) near/3 (adheren* OR complia* OR non-adheren* OR "non adheren*")) ) OR AB ( ((medication* OR drug* OR pharma*) near/3 (adheren* OR complia* OR non-adheren* OR "non adheren*")) )

# AND

( (MH "Chronic Disease+") OR (MH "Multiple Chronic Conditions") OR (MH "Long-Term Care") ) OR AB ( (("long term" or "chronic" or "longlasting or "persistant") near/3

(condition* or "disease" or "illness" or "sickness")) ) OR TI ( (("long term" or "chronic" or "longlasting or "persistant") near/3 (condition* or "disease" or "illness" or "sickness")) )

# AND

(( MH "digital intervention" or technology or internet or "mobile application") ) OR ( ( ((digital or "mobile app*" or technolog* or "internet") near/3 (intervention* or therap* or strateg*)) ) ) OR ( ( ((digital or "mobile app*" or technolog* or "internet") near/3 (intervention* or therap* or strateg*)) ) )

**Scopus**

(MH "Medication Adherence+") OR TI ( ((medication* OR drug* OR pharma*) w/3 (adheren* OR complia* OR non-adheren* OR "non adheren*")) ) OR AB ( ((medication* OR drug* OR pharma*) w/3 (adheren* OR complia* OR non-adheren* OR "non adheren*")) )

( (MH "Chronic Disease+") OR (MH "Multiple Chronic Conditions") OR (MH "Long-Term Care") ) OR AB ( (("long term" or "chronic" or "longlasting or "persistant") w/3 (condition* or "disease" or "illness" or "sickness")) ) OR TI ( (("long term" or "chronic" or "longlasting or "persistant") w/3 (condition* or "disease" or "illness" or "sickness")) )

# AND

(( MH "digital intervention" or technology or internet or "mobile application") ) OR ( ( ((digital or "mobile app*" or technolog* or "internet") w/3 (intervention* or therap* or strateg*)) ) ) OR ( ( ((digital or "mobile app*" or technolog* or "internet") w/3 (intervention* or therap* or strateg*)) ) )
