# Supplementary material for: Contexts and Mechanisms Related to the Efficacy of Digital Medication Adherence Interventions to Support Medication Adherence Among Adults With Chronic Diseases: Realist Review
Source: Interact J Med Res. 2026 Aug 28;15:e89803. doi: 10.2196/89803 (PMC13524365; doi:10.2196/89803)
Supplement: Supplementary file 3 [file ijmr-v15-e89803-s003.docx]

| **Authors & Year of publication** | **Study Title** | **Document type (review/commentary/ qualitative/quantitative/mixed method/ RCT/pre-post (intervention studies)** | **Participant characteristics and country in which study conducted (for primary studies)** | **Rigour (0-3)**  **Reasons for scores in case of scores between 0-2** | **Richness (0-4)**  **Reasons for scores in case of scores between 0-3** | **Relevance (0-3)**  **Reasons for scores in case of scores between 0-2** | **provided data for CMO theory number** |
| --- | --- | --- | --- | --- | --- | --- | --- |
| Wiecek et al (2020) [37] | Impact of a Multicomponent  Digital Therapeutic Mobile App  on Medication Adherence in Patients  with Chronic Conditions:  Retrospective Analysis | A retrospective  observational study | Country: Australia Any chronic disease population | 3 | 1: Provided adequate information about mechanisms but less detailed information about  contexts including participant characteristics that may influence medication adherence. The authors mentioned that they did not have information on the users’ clinical conditions.  Due to this, they were unable to perform sub-analyses  based on patient age, gender, medication, or condition. They also could not assess baseline adherence rates. | 1: Lack of contextual details. | CMO1 |
| Wang et al., (2024) [91] | Exploration of Features of  Mobile Applications for Medication  Adherence in Asia: Narrative Review | Narrative review | N/A | 2: Dates limited to  2019-2024 and no  reason has been provided for that. | 2: More detailed contextual  information would have made it clearer. | 2: More contextual information would have led to more information for building programme theories. | CMO 1, CMO17, |
| Gackowski et al., (2024) [52] | Innovative Approaches  to Enhance and Measure  Medication Adherence  in Chronic Disease  Management: A Review | Review | N/A | 1: Did not describe the methodology with which information was searched ie the databases, dates etc. | 2: More information  about contextual factors required especially ingestible biosensors (it was not clear why the authors  concluded that it could help in  improving the health of older adults and can give them the confidence to take charge of their health, especially considering the ethical issues with implementing them) | 2: Provided adequate information about mechanisms  but details about contexts were lacking for some  devices | CMO1, CMO8, CMO 15, |
| Pouls et al., (2021) [51] | Effect of Interactive eHealth  Interventions on Improving  Medication Adherence in Adults  With Long-Term Medication:  Systematic Review | systematic review | N/A | 3 | 2: not much contextual information | 2: Lack of contextual information of the included studies. | CMO1 |
| Peng et al., (2020) [50] | Effectiveness of Mobile Applications  on Medication Adherence in Adults  with Chronic Diseases: A Systematic  Review and MetaAnalysis | systematic review | N/A | 2: Methodology was rigorous except that it was not clear how analytic hierarchy process of each feature of the app was calculated and the clear data including p values, effect sizes of each individual intervention effectiveness was not provided. | 1: Not very detailed explanation of  mechanisms outcomes and contexts  were discussed. Pooled subgroup analysis  of the app features were done which may not be informative as these are complex interventions and each component of the intervention interacts with  others to bring about the outcomes. Pooled efficacy of  all the studies were assessed but mobile app interventions are likely to be heterogenous in terms of content and delivery. | 1: Not very relevant information for forming programme theories | CMO1 |
| Arain et al., (2021) [31] | Medication adherence support  of an in-home electronic medication  dispensing system for individuals  living with chronic conditions: a pilot  randomized controlled trial | RCT | Country: Canada Any chronic condition | 3 | 3: Further exploration of contextual factors, e.g. acceptability of the intervention or variation in efficacy due to certain demographic factors would have been better. | 2: More contextual details would have  led to more information about CMO  theory development | CMO1, CMO15 |
| Donovan et al., (2022) [107] | Influencing medication taking behaviours  using automated two‐way digital  communication: A narrative synthesis  systematic review informed by  the Behaviour Change Wheel | systematic review | n/a | 3 | 4 | 3 | CMO8, CMO 13 |
| Badawy et al., (2020) [87] | Habit Strength, Medication Adherence,  and Habit-Based Mobile Health Interventions  Across Chronic Medical Conditions: Systematic Review | systematic review | n/a | 1: one database searched only and no quality  assessment of studies | 2: Lot of detailed information about contexts  but not mechanisms of the apps to increase habit strength. | 2: Not a lot of information on mechanisms to improve habit strength. | CMO1, CMO2 |
| Espírito-Santo et al., (2024) [101] | Digital Solutions Available  to Be Used by Informal Caregivers , Contributing to Medication  Adherence: A Scoping Review | scoping review | for informal carers n/a | 3 | 4 | 3 | CMO15 |
| Linn et al., (2011) [11] | Effects of eHealth Interventions  on Medication Adherence: A  Systematic Review of the Literature | systematic review | n/a | 3 | 2: more detailed information about contextual factors would have been more useful. | 2: Not enough detail about contextual factors hence there was less information for CMO theory development | CMO 8 |
| Mira et al., (2014) [32] | A Spanish Pillbox App  for Elderly Patients  Taking Multiple Medications : Randomized Controlled Trial | RCT | Country: Spain Patients with multiple chronic  diseases, older than 60 years, multiple medications | 3 | 4 | 3 | CMO1, CMO8, CMO 9 |
| He et al., (2022) [33] | Application of family-involved  smart medication management  system in rural-dwelling middle-aged  and older adult participants with  chronic diseases: Management  of chronic diseases in rural areas | RCT | Country: China,  More than 45 years of age  diagnosed with any chronic disease | 3 | 4 | 3 | CMO1, CMO 15 |
| Park et al., (2017) [40] | Implications for Training  on Smartphone Medication  Reminder App Use by Adults  With Chronic Conditions: Pilot  Study Applying the Technology  Acceptance Model | Pilot intervention study | Country: USA Any chronic disease middle aged and older adults above age of 45 years. | 3 | 4 | 3 | CMO 1, CMO 5, CMO 9, CMO17, CMO 22 |
| Russell et al., (2018) [45] | Older Adult Preferences  of Mobile Application  Functionality Supporting  Medication Self-Management | Qualitative | Country: USA Older adults above 55 years age, 5 or more prescription meds and multiple chronic diseases. | 3 | 4 | 3 | CMO 1, CMO 8 |
| Park et al., (2014) [90] | A quantitative systematic  review of the efﬁcacy of  mobile phone interventions  to improve medication adherence | systematic review | n/a | 3 | 2: More details about contextual factors  would have been better | 2: Insufficient contextual information. | CMO1 |
| Andrikopoulou et al., (2019) [55] | What are the important  design features of personal  health records to improve  medication adherence for  patients with long-term  conditions? A systematic  literature review | systematic review | n/a | 3 | 3: More details about contextual factors  would have been better | 2: Insufficient contextual information. | CMO1, CMO 8 |
| Zhou et al., (2022) [53] | Effectiveness of Mobile Medical  Apps in Ensuring Medication  Safety Among Patients With  Chronic Diseases: Systematic  Review and Meta-analysis | systematic review | n/a | 1: Meta-analysis conducted with  certain studies and no clear rationale  about the studies included in the  meta-analysis and in case of medication  adherence, no information about what  studies were included in the meta-analysis  for medication adherence. In the information  about efficacy about individual studies, there was no information about p-values and/or effect sizes. | 1: Very limited information about  mechanisms and contexts related to effectiveness to improve medication safety and adherence | 2: Limited information about app  features effective in specific contexts to  improve medication adherence and safety | CMO1 |
| Reidel et al., (2008) [38] | Pilot study of an  interactive voice  response system to improve medication refill compliance | Qualitative: feasibility study | Country: Canada Any chronic condition | 2: No information about the type of qualitative data analysis used and the ways researchers improved the credibility, transferability, dependability and confirmability of the research. | 4 | 3 | CMO1, CMO 26 |
| Miguel-Cruz et al (2019) [56] | What does the literature  say about using electronic  pillboxes for older adults?  A systematic literature review | systematic review | n/a, for older adults | 3 | 2: More detailed information on features of the pillboxes and contextual factors  would have been better. | 2: Insufficient information about the mechanisms. | CMO 1, CMO 8 |
| Armitage et al  (2020) [9] | Do mobile device apps  designed to support medication  adherence demonstrate efficacy?  A systematic review of randomised  controlled trials, with meta-analysis. | systematic review | n/a | 2: The pooling of data from  a group of studies which used a variety of methods for measurement of medication adherence and targeted at patients with different healthcare problems. BCTs can be delivered in different ways and content could be different as well and a narrative review would have been better suited. | 1: limited information about mechanisms  and contexts related to efficacy/non-effectiveness of the apps | 1: limited information about mechanisms  and contexts related to efficacy/non-effectiveness of the apps | CMO 2, CMO 8 |
| Goradia et al., (2021) [89] | A new age  intervention to  support medication adherence | review | n/a | 3 | 4 | 3 | CMO 1, CMO 8 |
| Colon et al., (2020) [58] | Patient Assistance Programs  and Technology in Medication Adherence | review | n/a | 3 | 1: limited details about mechanisms of technological interventions that can help improve medication  adherence | 2: Limited detail about contextual factors and mechanisms, hence limited information for programme theory development. | CMO1 |
| Jiménez-Chala et al., (2022) [92] | Use of Mobile Applications  to Increase Therapeutic  Adherence in Adults:  A Systematic Review. | systematic review | n/a | 3 | 1: limited details about mechanisms of technological interventions that can help improve medication  adherence | 1: Limited detail about contextual factors and mechanisms | CMO 1 |
| Anglada-Martinez  et al (2015) [10] | Does mHealth increase  adherence to medication?  Resultsof a systematic review | systematic review | n/a | 3 | 2: less details about relationships between contexts and mechanisms of the interventions | 1: Limited detail about contextual factors and mechanisms | CMO1 |
| Emadi et al., (2022) [57] | Factors affecting  medication adherence  among older adults  using tele-pharmacy  services: a scoping  review. | scoping review | n/a for older adults above 60 years | 3 | 4 | 3 | CMO 1, CMO 15 |
| Ahmad et al (2020) [42] | Perceptions of an  in-Home Electronic  Medication Dispensing  System: A Qualitative Study. | Qualitative | Country: Canada above 50 years age, any chronic condition taking 5 or more oral meds | 3 | 4 | 3 | CMO1, CMO 15, CMO 16 |
| Castillo et al., (2021) [39] | Digital medicines for  adherence support:  A conceptual framework  and qualitative study  of adherence among  chronically ill patients | Qualitative | Country: USA  taking one or more prescribed medication  for diabetes, hypertension, or high cholesterol be identified, by their providers, as having high disease  risk, as reflected in elevated A1C (blood test that measures  average blood glucose level over the past 3 months), blood  pressure, or low-density lipoprotein levels (measure of harmful cholesterol levels). | 3 | 4 | 3 | CMO 1, CMO2, CMO8 |
| Sarzynski et al., (2017) [29] | Beta Testing a Novel  Smartphone Application  to Improve Medication Adherence | 2 phase Feasibility &  acceptability study | Country: Not reported any chronic condition | 2: Quantitative measures used only on small population more in-depth qualitative design would have been more suitable | 1: very less information about contexts  and mechanisms related to efficacy of the app | 1: Article was mostly focussed on the software development of the app and very less information was present about the mechanisms by which  the app helped in improving adherence and there was limited contextual information. | CMO 1 |
| Eggerth et al (2020) [59] | Medication management  needs information and  communications technology‐based approaches,  including telehealth and artificial intelligence | review | n/a | 3 | 2: More contextual information related to tele-heath effectiveness would have been better. | 2: Further contextual details would be better. | CMO1, CMO 8 |
| Hincapie et al., (2019) [46] | Exploring Perceived Barriers  to Medication Adherence  and the Use of Mobile Technology  in Underserved Patients With  Chronic Conditions | Qualitative | Country: USA had a diagnosis of at least one of the following  chronic disease states: diabetes, dyslipidemia,  and/or hypertension and had been prescribed 3  long-term medications for at least 3 months. Recruited from areas with limited access to primary care providers, high infant mortality, high poverty, and/or high older adult population | 3 | 4 | 3 | CMO1, CMO 8, CMO 24 |
| Faisal et al., (2022) [34] | Integration of a smart  multidose blister package  for medication intake: A  mixed method ethnographic  informed study of older adults  with chronic diseases | Mixed methods study | Country: Canada older adults had more than one chronic disease,  (3) on a complex medication regimen (defined as  taking five or more oral medications per day, or if taking less than five oral medications per day, taking a more than once-daily dosing schedule for an oral medication), self-managing their medications regularly | 3 | 4 | 3 | CMO1, CMO 15, CMO 16 |
| Shahani et al., (2022) [35] | An electronic pillbox intervention  designed to improve medication  safety during care transitions:  challenges and lessons learned  regarding implementation and evaluation | cluster-randomized controlled trial | Country: USA Recently discharged patients from  hospital. 5 or more chronic medications | 3 | 4 | 3 | CMO1, CMO 3, CMO 4, CMO 10, CMO 11, CMO 15, CMO 28 |
| Hersberger et al (2013) [60] | Dose-dispensing service  as an intervention to  improve adherence to polymedication | opinion paper | n/a | 3 | 2: More contextual information related to effectiveness would have been better | 2: limited contextual information,  hence less information for theory development | CMO1, CMO 15 |
| Anglada-Martínez et al., (2016) [43] | Feasibility and Preliminary Outcomes  of a Web and Smartphone–Based  Medication Self-Management Platform  for Chronically Ill Patients | single-arm prospective pre-post  intervention study | Country: Spain receiving treatment for hypertension  and/or dyslipidemia and/or heart failure  and/or human immunodeficiency virus infection | 3 | 2: More detailed contextual information related to effectiveness would have been better | 2: Limited information about contextual factors related to effectiveness. | CMO1 |
| Billingsley and Carruth (2015) [61] | Use of Technology to Promote  Effective Medication Adherence. | review | n/a | 3 | 2: More contextual information  would have been better | 2: Lacked information about contexts in which the technologies could be effective/non-effective | CMO1, CMO15 |
| Brondi et al., (2013) [30] | Drugs Don’t Work in Patients Who  Don’t Take Them: Dr. Drin, The  New ICT Paradigm for Chronic Therapies | feasibility study | Country: Not reported any chronic condition | 1: the psychometric validity and reliability of the measures used have not been described.  Only preliminary data from 12 participants presented so it was not possible to ascertain if there was any significant improvement from the baseline. | 1: More contextual information would have  been better | 1: Lacked contextual information about effectiveness/non-effectiveness because of which less information for theory development | CMO 1, CMO 2 |
| Bakema et al., (2023) [93] | The Use of eHealth for Pharmacotherapy  Management with Patients with Respiratory  Disease, Cardiovascular Disease, or Diabetes: Scoping Review | scoping review | n/a patients with respiratory disease,  cardiovascular disease or diabetes | 3 | 2: More contextual information  would have been better | 2: Lacked information about contexts in which the technologies could be effective/non-effective | CMO1, CMO 8 |
| Thakkar et al., (2016) [62] | Mobile Telephone Text Messaging  for Medication Adherence in Chronic  Disease: A Meta-analysis | meta-analysis | n/a | 2: The pooling of data from  a group of studies which used a variety of methods for measurement of medication adherence and targeted at patients with different healthcare problems. A narrative review would have probably provided more detailed information about contexts and mechanisms. | 1: Meta-analysis was conducted  because of which there was lack of detailed information about the mechanisms via which interventions could be effective and contexts in which they can be effective | 1: Lacked contextual information about effectiveness/non-effectiveness because of which less information for theory development | CMO1 |
| Schneider et al  (2013) [88] | Cyberhealth serving to support  individual intake of medication | Review | n/a | 3 | 4 | 3 | CMO 1, CMO 8, CMO 15 |
| Tolley et al., (2023) [105] | Interventions to promote medication adherence for chronic diseases in India: a systematic review | systematic review | n/a interventions only in India | 3 | 2: Some more detailed information on the  components of the technological interventions would have been better as that would have helped to understand the mechanisms better. More contextual information also would have been better. | 2: Less detail about mechanisms of interventions hence there was less information for CMO theory development | CMO 24 |
| Lammila-Escalera et al., (2024) [94] | Interventions to improve medication  adherence in adults with mental-physical  multimorbidity in primary care: a systematic review. | systematic review | n/a only patients with comorbid physical mental health conditions | 3 | 1: Limited details about the ICT studies which made it difficult to understand the mechanisms and contexts related to their efficacy/non-effectiveness | 1: very limited details about ICT intervention mechanisms and contextual factors hence there was less information for CMO theory development | CMO1 |
| Hugtenburg et al., (2013) [63] | Definitions, variants, and causes  of nonadherence with medication:  a challenge for tailored interventions | Review | n/a | 3 | 4 | 3 | CMO1, CMO 8 |
| Liu and Varshney (2020) [64] | Mobile health: A carrot and stick intervention to improve  medication adherence | Review & analytical & theoretical modeling | n/a | 2: The intervention effectiveness was assessed using probability data only.  However, the methodology of the study was strengthened by the review of medication adherence interventions conducted that also provided information about CMOs. | 4 | 3 | CMO1, CMO 15 |
| Marengoni et al., (2016) [65] | Strategies to Improve Medication  Adherence in Older Persons: Consensus  Statement from the Senior Italia Federanziani Advisory Board | opinion paper | n/a focussed on older people | 3 | 4 | 3 | CMO1, CMO 15 |
| Bingham et al., (2021) [66] | Impact of Telehealth Interventions on Medication Adherence for Patients With  Type 2 Diabetes, Hypertension, and/or Dyslipidemia: A Systematic Review | systematic review | n/a, only Patients With  Type 2 Diabetes, Hypertension, and/or Dyslipidemia | 3 | 2: More contextual information about effectiveness/non-effectiveness of interventions would have been better | 2: Lacked adequate contextual details. | CMO1 |
| Wiecek et al., (2019) [54] | Temporal effectiveness of interventions  to improve medication adherence: A network meta-analysis | meta-analysis | n/a | 2: One database searched so evidence might be limited. | 2: More contextual information about temporal effectiveness would have been better | 2: Less detail about contextual factors hence there was less information for CMO theory development | CMO1, CMO 8 |
| LaValley et al., (2023) [47] | Family Caregivers of Older Adults:  Strategies, Tools, and Technologies  for Medication Management | Qualitative | Country: USA Informal caregivers of older adults above  65 years age | 3 | 4 | 3 | CMO 25 |
| Fallah and Yasini (2017) [36] | A Medication Reminder Mobile App:  Does It Work for Different Age Ranges | feasibility study | country not reported. No information about medications, specific age range  or illness diagnoses of participants | 1: no sample size calculation and authors acknowledged  that sample size was very low. The psychometric properties  of the questionnaires used to assess the usability and  perceived efficacy is not described. | 1: Very less information about contexts and the participants who were recruited to assess usability and perceived efficacy. Only age was reported. Mechanisms by which the app aims to enhance adherence is described in less details as well | 1: Very limited details about mechanisms and contextual factors hence there was less information for CMO theory development | CMO1 |
| Kardas et al., (2022) [67] | Reimbursed medication adherence  enhancing interventions in 12 European  countries: Current state of the art and future challenges | review | n/a | 3 | 2: Particular contextual factors in each country are limited | 2: limited contextual information. | CMO1 |
| Donovan et al., (2022) [48] | Two-way Automated Text Messaging  Support from Community Pharmacies  for Medication Taking in Multiple Long-term Conditions:  Human-Centered Design With Nominal Group Technique Development Study | Qualitative | country: UK Healthcare professionals and patients with any chronic disease | 2: No information about the ways researchers improved  the credibility, transferability, dependability and confirmability of the research | 4 | 3 | CMO 1, CMO 8, CMO 13 |
| Doggrell (2010) [95] | Adherence to medicines in the older-aged  with chronic conditions – does an intervention  concerning adherence by an allied health professional help? | review | n/a older adults focussed | 3 | 4 | 3 | CMO1, CMO 2, CMO 15 |
| Balasubramanian et al (2021) [41] | Digital personal assistants are smart ways  for assistive technology to aid the health  and wellbeing of patients and carers | Service evaluation/market research using mixed methods. | Country: UK. The age of the  study participants ranged from 50 to 90 years and both informal carers and patients. | 2: Ways to increase credibility, dependability, transferability of qualitative data not reported. | 4 | 3 | CMO 1, CMO 18 |
| Backes et al., (2020) [49] | Digital Medication Adherence Support:  Could Healthcare Providers Recommend Mobile Health Apps? | quantitative survey study | Country: Switzerland patients between 30-60 years of age and taking meds for more than 10 years. | 1: Psychometric properties of the questionnaire used to test the patients' evaluation of the apps  not mentioned. Details of if it included open or close ended questions not reported. No sample size calculation. | 2: less contextual details | 2: Less detail about contextual factors hence there was less information for CMO theory development | CMO1 |
| Lancaster et al., (2018) [98] | The use and effects of e-health tools  for patient self-monitoring and reporting  of outcomes following medication use: A systematic review | systematic review | n/a | 3 | 2: less details about contexts related to effectiveness/non-effectiveness of interventions | 2: Less detail about contextual factors hence there was less information for CMO theory development | CMO 8 |
| Hartch et al., (2023) [3] | Effect of a Medication Adherence Mobile Phone App on Medically Underserved Patients with Chronic Illness: Preliminary Efficacy Study | single-arm pre-post intervention preliminary efficacy study | Country: USA Adults with any chronic illness | 3 | 4 | 3 | CMO1, CMO 8 |
| Torres-Robles et al., (2018) [68] | Comparison of Interventions to Improve  Long-Term Medication Adherence Across  Different Clinical Conditions: A Systematic  Review with Network Meta-Analysis | meta-analysis | n/a | 3 | 4 | 3 | CMO1, CMO 13 |
| Tantray et al., (2024) [69] | Prescription Precision: A Comprehensive  Review of Intelligent Prescription Systems | review | n/a | 3 | 2: less details about contexts related to effectiveness/non-effectiveness of interventions | 1: Less detail about contextual factors hence there was less information for CMO theory development | CMO1 |
| Schneider et al., (2009) [102] | Adherence: a review of education,  research, practice and policy in Switzerland | review | n/a | 3 | 2: Lack of details regarding mechanisms and intervention components and contexts related to efficacy/non-efficacy of interventions | 2: Lack of details regarding the mechanisms of the interventions and contexts related to efficacy/non-efficacy of interventions. Thus, less information for CMO theory development. | CMO 15 |
| Sun et al., (2024) [70] | Mobile phone interventions to improve  health outcomes among patients with chronic  diseases: an umbrella review and evidence synthesis from 34 meta-analyses | umbrella review of meta-analysis | n/a | 3 | 1: Lack of details regarding mechanisms and intervention components and contexts related to efficacy/non-efficacy of interventions | 1: Lack of details regarding the mechanisms of the interventions and intervention components and contexts related to efficacy/non-efficacy of interventions. Thus, less information for CMO theory development. | CMO1 |
| Yasmin et al., (2016) [104] | Positive influence of short message service  and voice call interventions on adherence  and health outcomes in case of chronic disease care: a systematic review | systematic review | n/a | 3 | 2: More detailed information about contexts and mechanisms related to efficacy of interventions required. | 2: Lacked details about contexts and mechanisms, so less information for CMO development | CMO 24 |
| Pirri et al (2020) [71] | Scoping review and bibliometric analysis  of Big Data applications for Medication adherence:  an explorative methodological study to enhance consistency in literature | scoping review | n/a | 3 | 2: More detailed information about contexts and mechanisms related to efficacy of interventions required. | 2: Lacked details about contexts and mechanisms, so less information for CMO development | CMO1 |
| Stecher et al., (2024) [44] | Cost Savings From an mHealth Tool  for Improving Medication Adherence | intervention study (Quasi experimental) | country: USA people with chronic conditions like COPD, Asthma, depression, bipolar, schizophrenia, opioid disorder and low adherence over last 12 months. | 3 | 1: less details about contexts related to effectiveness/non-effectiveness of interventions. Other demographic details could have been assessed as covariates of the intervention effectiveness to explore contextual factors affecting effectiveness/non-effectiveness of the intervention. | 1: Less detail about contextual factors hence there was less information for CMO theory development | CMO 1 |
| Schwartz et al., (2017) [72] | Intervention to Improve Medication  Management: Qualitative Outcomes  From a Phase I Randomized Controlled Trial | Qualitative | Country: USA  adults diagnosed with a chronic health condition,  on a medication regimen of ﬁve or more medications,  who live in the community and independently manage their medications. | 3 | 1: There were lack of details regarding the assistive  technology that were recommended to participants. Contextual information was lacking as well. | 1: Lack of details regarding the assistive technology recommended or provided to participants  led to lesser information for CMO theory development | CMO1 |
| Haugh (2014) [73] | Medication adherence in older adults : the pillbox half full. | Review | n/a focussed on older people | 3 | 4 | 3 | CMO1, CMO 8, CMO 15 |
| Zullig et al (2014) [74] | Applying Technology to Medication  Management and Adherence | review | n/a | 3 | 4 | 3 | CMO1, CMO 16, CMO 18, |
| Austin and Hull (2014) [75] | The power of mobile health technologies  and prescribing apps | opinion paper | n/a | 3 | 1: Lack of details regarding the mechanisms and contexts. | 1: Lack of details regarding the mechanisms of the interventions and contexts related to efficacy/non-efficacy of interventions. Thus, less information for CMO theory development. | CMO1 |
| Aungst (2021) [99] | Reevaluating medication adherence  in the era of digital health | review | n/a | 3 | 4 | 3 | CMO 13, CMO 15 |
| Choi et al., (2020) [96] | Mobile-Application-Based Interventions  for Patients with Hypertension and Ischemic  heart disease: A Systematic Review | systematic review | n/a focussed on Patients with Hypertension and Ischemic  heart disease | 3 | 2: More details regarding mechanisms and intervention components and contexts related to efficacy/non-efficacy of interventions would be better. | 2: More details regarding the mechanisms of the interventions and contexts would have been better for programme theory development | CMO1, CMO8 |
| Viswanathan et al., (2012) [76] | 4. Medication Adherence Interventions : Comparative Effectiveness | evidence report of a systematic  review | n/a | 3 | 1: Lack of details regarding mechanisms and intervention components and contexts related to efficacy/non-efficacy of interventions. This is because of lack of standardised reporting of the details about intervention components in the included studies in the review acknowledged by the authors. | 1: Lack of details regarding the mechanisms of the interventions and contexts related to efficacy/non-efficacy of interventions. Thus, less information for CMO theory development. | CMO1 |
| Mash et al (2001) [100] | Alternative mechanisms for delivery  of medication in South Africa: A scoping review | scoping review | n/a | 3 | 1: Lack of detailed information about contexts and mechanisms  related to efficacy/non-efficacy of digital or technology enabled drug dispensing interventions like smart lockers or pharmacy dispensing units. | 1: Lack of detailed information about contexts and mechanisms  related to efficacy/non-efficacy of digital or technology enabled drug dispensing interventions like smart lockers or pharmacy dispensing units. Thus, less information for CMO theory development. | CMO 14 |
| Aranda-Jan et al (2014) [77] | Systematic review on what works,  what does not work and why of implementation  of mobile health (mHealth) projects in Africa | systematic review | n/a | 3 | 2: Lack of details regarding mechanisms and intervention components and contexts related to efficacy/non-efficacy of the interventions that aimed to improve medication adherence. | 2: Lack of details regarding mechanisms and intervention components and contexts related to efficacy/non-efficacy of the interventions that aimed to improve medication adherence. | CMO1, CMO 14 |
| United States Government Accountability Office & national academy of medicine (2022) [97] | Artificial Intelligence in Health Care Benefits and Challenges of Technologies to Augment Patient Care | report | n/a | 3 | 1: Lack of details regarding mechanisms and intervention components and contexts related to efficacy/non-efficacy of the interventions that aimed to improve medication adherence. | 1: Lack of details regarding mechanisms and intervention components and contexts related to efficacy/non-efficacy of the interventions that aimed to improve medication adherence. Thus, less information for CMO theory development. | CMO 1 |
| Academy of Medical Sciences (2015) [78] | Patient adherence to medicines | report | n/a | 3 | 4 | 3 | CMO1, CMO 15 |
| Khan and Socha-Dietrich (2018) [79] | Investing in medication adherence improves health outcomes and health system efficiency:  Adherence to medicines for diabetes, hypertension, and hyperlipidaemia | working paper | n/a focussed on diabetes, hypertension,  and hyperlipidaemia | 3 | 4 | 3 | CMO1, CMO 8, CMO 13 |
| Australian Government Department of Health and Aged Care (2024) [80] | Health Technology Assessment Policy and Methods Review: Emerging health technologies | report | n/a | 3 | 2: Lack of adequate details regarding mechanisms and intervention components and contexts related to efficacy/non-efficacy of the interventions that aimed to improve medication adherence. | 2: More details regarding mechanisms and intervention components and contexts would have been better. | CMO1, CMO 13 |
| Perry et al., (2015) [103] | Behavioural insights in health care: Nudging to reduce inefficiency and waste | report | n/a | 3 | 1: Lack of details regarding mechanisms and intervention components and contexts related to efficacy/non-efficacy of the interventions that aimed to improve medication adherence. | 1: Lack of details regarding mechanisms and intervention components and contexts related to efficacy/non-efficacy of the interventions that aimed to improve medication adherence. Thus, less information for CMO theory development. | CMO 15 |
| World Health Organization (2019) [81] | Medication Safety in Polypharmacy | Technical report | n/a | 3 | 2: Lack of details regarding mechanisms and intervention components and contexts related to efficacy/non-efficacy of some of the interventions. For example: there was lack of details about the types of patients and medication formulations that would not be suitable for pill dispensers, dose administration aids and multi component medication compliance devices. | 2: Further of details regarding mechanisms and intervention components and contexts would have been better. | CMO1, CMO 15 |
| Cross et al., (2020) [82] | Interventions for improving medication-taking  ability and adherence in older adults prescribed multiple medications | meta-analysis | n/a focussed on older people prescribed with multiple meds | 2: Adherence interventions are complex and categorizing them into  educational, behavioural and mixed behavioural and educational interventions  hindered the understanding of the intervention mechanisms of some studies. | 2: Less details about contexts and mechanisms related to efficacy or non-efficacy of interventions. Poor description of usual care  control conditions. Authors also noted variation in the types of medications reported across studies, with some reporting only prescription medication, some reporting adherence to only regular medication, and some reporting all medications (e.g. regular, when required, prescription, nonprescription medications). | 2: Lack of details regarding mechanisms and  intervention components and contexts related  to efficacy/non-efficacy of the interventions. | CMO1, CMO 15 |
| Nieuwlaat et al., (2014) [83] | Interventions for enhancing medication adherence (Review) | systematic review | n/a | 3 | 2: Lack of details regarding mechanisms and intervention components and contexts. Authors acknowledge that inconsistency in assessing medication adherence across studies and lack of detailed information about both intervention and control arms contributed to difficulty in understanding the mechanisms. | 2: Lack of details regarding mechanisms and intervention components  and contexts. | CMO1, CMO 15 |
| Coulter and Ellins (2006) [84] | Patient-focused interventions: A review  of the evidence | report | n/a | 3 | 1: Lack of details regarding mechanisms and intervention components and contexts related to efficacy/non-efficacy of the interventions. | 1: Lack of details regarding mechanisms and intervention components and contexts related to efficacy/non-efficacy of the interventions that aimed to improve medication adherence. Thus, less information for CMO theory development. | CMO1 |
| International longevity centre UK (2022) [85] | Doctor’s Orders  Why adherence is critical to improving health outcomes across the life course | report | n/a | 3 | 4 | 3 | CMO1, CMO 8, CMO 26 |
| NICE (2023) [86] | YOURmeds for medication support in long-term conditions | report | n/a | 1: The report does state that evidence for the technology was relatively low and that further larger  studies with comparator groups were needed. Expert opinions such as those of community pharmacists were discussed in this report but the research methodology used to elicit them is not clear. | 4 | 3 | CMO1, CMO 8, CMO 15 |
| Posadzki et al., (2016) [106] | Automated telephone communication  systems for preventive healthcare and management of long-term conditions (Review) | systematic review | n/a | 3 | 2: Lack of details regarding mechanisms and intervention components and contexts related to efficacy/non-efficacy of the interventions that aimed to improve medication adherence. Authors acknowledge that lack of detailed information about treatment arms, contributed to difficulty in understanding the mechanisms of interventions that led to efficacy/non-efficacy. a large proportion of studies did not report whether or not participants received instructions on how to use the system. In several trials, it was unclear whether patients or healthcare professionals initiated the calls or whether (or not) the participants used a telephone keypad to interact with the systems. Information about the intervention's duration, frequency and intensity; safety (adverse eﬀects) and cost-eﬀectiveness; security arrangements; or speakers' features was frequently missing, too. | 2: Lack of details regarding mechanisms and intervention components  and contexts related to efficacy/non-efficacy of the interventions that aimed to improve medication adherence. Thus, less information for CMO theory development. | CMO26 |

**References:**

37. Wiecek E, Torres-Robles A, Cutler RL, Benrimoj SI, Garcia-Cardenas V. Impact of a multicomponent digital therapeutic mobile app on medication adherence in patients with chronic conditions: retrospective analysis. J Med Internet Res. Aug 12, 2020;22(8):e17834. [doi: 10.2196/17834] [Medline: 32784183]

91. Wang T, Huang YM, Chan HY. Exploration of features of mobile applications for medication adherence in Asia: narrative review. J Med Internet Res. Nov 8, 2024;26:e60787. [doi: 10.2196/60787] [Medline: 39514859]

52. Gackowski M, Jasińska-Stroschein M, Osmałek T, Waszyk-Nowaczyk M. Innovative approaches to enhance and measure medication adherence in chronic disease management: a review. Med Sci Monit. Jul 16, 2024;30:e944605. [doi: 10.12659/MSM.944605] [Medline: 39012851]

51. Pouls BPH, Vriezekolk JE, Bekker CL, et al. Effect of interactive eHealth interventions on improving medication adherence in adults with long-term medication: systematic review. J Med Internet Res. Jan 8, 2021;23(1):e18901. [doi: 10.2196/18901] [Medline: 33416501]

50. Peng Y, Wang H, Fang Q, et al. Effectiveness of mobile applications on medication adherence in adults with chronic diseases: a systematic review and meta-analysis. J Managed Care Spec Pharm. Apr 2020;26(4):550-561. [doi: 10.18553/ jmcp.2020.26.4.550] [Medline: 32223596]

31. Arain MA, Ahmad A, Chiu V, Kembel L. Medication adherence support of an in-home electronic medication dispensing system for individuals living with chronic conditions: a pilot randomized controlled trial. BMC Geriatr. Jan 14, 2021;21(1):56. [doi: 10.1186/s12877-020-01979-w] [Medline: 33446126]

107. Donovan G, Hall N, Ling J, Smith F, Wilkes S. Influencing medication taking behaviors using automated two-way digital communication: A narrative synthesis systematic review informed by the Behavior Change Wheel. Br J Health Psychol. 2022 Sep;27(3):861-890. doi: 10.1111/bjhp.12580. Epub 2022 Jan 26. PMID: 35080811; PMCID: PMC9541766.

87. Badawy SM, Shah R, Beg U, Heneghan MB. Habit strength, medication adherence, and habit-based mobile health interventions across chronic medical conditions: systematic review. J Med Internet Res. Apr 28, 2020;22(4):e17883. [doi: 10.2196/17883] [Medline: 32343250]

101. Espírito-Santo M, Santos S, Estêvão MD. Digital solutions available to be used by informal caregivers, contributing to medication adherence: a scoping review. Pharmacy (Basel). Jan 23, 2024;12(1):20. [doi: 10.3390/pharmacy12010020] [Medline: 38392927]

11. Linn AJ, Vervloet M, van Dijk L, Smit EG, Van Weert JCM. Effects of eHealth interventions on medication adherence: a systematic review of the literature. J Med Internet Res. Dec 5, 2011;13(4):e103. [doi: 10.2196/jmir.1738] [Medline: 22138112]

32. Mira JJ, Navarro I, Botella F, et al. A Spanish pillbox app for elderly patients taking multiple medications: randomized controlled trial. J Med Internet Res. Apr 4, 2014;16(4):e99. [doi: 10.2196/jmir.3269] [Medline: 24705022]

33. He J, Chen J, Li Q, et al. Application of family-involved smart medication management system in rural-dwelling middle-aged and older adult participants with chronic diseases: management of chronic diseases in rural areas. Medicine (Baltimore). 2022;101(45):e31662. [doi: 10.1097/MD.0000000000031662]

40. Park DY, Goering EM, Head KJ, Bartlett Ellis RJ. Implications for training on smartphone medication reminder app use by adults with chronic conditions: pilot study applying the technology acceptance model. JMIR Form Res. Nov 10, 2017;1(1):e5. [doi: 10.2196/formative.8027] [Medline: 30684397]

45. Russell AM, Smith SG, Bailey SC, et al. Older adult preferences of mobile application functionality supporting medication self-management. J Health Commun. 2018;23(12):1064-1071. [doi: 10.1080/10810730.2018.1554728] [Medline: 30526400]

90. Park LG, Howie-Esquivel J, Dracup K. A quantitative systematic review of the efficacy of mobile phone interventions to improve medication adherence. J Adv Nurs. Sep 2014;70(9):1932-1953. [doi: 10.1111/jan.12400] [Medline: 24689978]

55. Andrikopoulou E, Scott P, Herrera H, Good A. What are the important design features of personal health records to improve medication adherence for patients with long-term conditions? A systematic literature review. BMJ Open. Sep 26, 2019;9(9):e028628. [doi: 10.1136/bmjopen-2018-028628] [Medline: 31558449]

53. Zhou TT, Wang R, Gu SJ, et al. Effectiveness of mobile medical apps in ensuring medication safety among patients with chronic diseases: systematic review and meta-analysis. JMIR mHealth uHealth. Nov 22, 2022;10(11):e39819. [doi: 10. 2196/39819] [Medline: 36413386]

38. Reidel K, Tamblyn R, Patel V, Huang A. Pilot study of an interactive voice response system to improve medication refill compliance. BMC Med Inf Decis Making. Oct 9, 2008;8:46. [doi: 10.1186/1472-6947-8-46] [Medline: 18845004]

56. Miguel-Cruz A, Bohórquez AF, Parra PAA. What does the literature say about using electronic pillboxes for older adults? A systematic literature review. Disabil Rehabil Assistive Technol. Nov 2019;14(8):776-787. [doi: 10.1080/ 17483107.2018.1508514] [Medline: 30451543]

9. Armitage LC, Kassavou A, Sutton S. Do mobile device apps designed to support medication adherence demonstrate efficacy? A systematic review of randomised controlled trials, with meta-analysis. BMJ Open. Jan 30, 2020;10(1):e032045. [doi: 10.1136/bmjopen-2019-032045] [Medline: 32005778]

89. Goradia S, Holland R, Alexander S, Greenbaum D, Chen T, Aslani P. A new age intervention to support medication adherence. Res Soc Administrative Pharm. Jun 2021;17(6):1204-1207. [doi: 10.1016/j.sapharm.2020.07.038] [Medline: 32811756]

58. Colon C, Salas P, Díaz M, et al. Patient assistance programs and technology in medication adherence. Innovations Pharm. 2020;11(2):10. [doi: 10.24926/iip.v11i2.3269] [Medline: 34007605]

92. Jiménez-Chala EA, Durantez-Fernández C, Martín-Conty JL, Mohedano-Moriano A, Martín-Rodríguez F, PolonioLópez B. Use of mobile applications to increase therapeutic adherence in adults: a systematic review. J Med Syst. Oct 22, 2022;46(12):87. [doi: 10.1007/s10916-022-01876-2] [Medline: 36272023]

10. Anglada-Martinez H, Riu-Viladoms G, Martin-Conde M, Rovira-Illamola M, Sotoca-Momblona JM, Codina-Jane C. Does mHealth increase adherence to medication? Results of a systematic review. Int J Clin Pract. Jan 2015;69(1):9-32. [doi: 10.1111/ijcp.12582] [Medline: 25472682]

57. Emadi F, Ghanbarzadegan A, Ghahramani S, Bastani P, Baysari MT. Factors affecting medication adherence among older adults using tele-pharmacy services: a scoping review. Arch Public Health. Aug 31, 2022;80(1):199. [doi: 10.1186/ s13690-022-00960-w] [Medline: 36042508]

42. Ahmad A, Chiu V, Arain MA. Users’ perceptions of an in-home electronic medication dispensing system: a qualitative study. Med Devices (Auckl). 2020;13:31-39. [doi: 10.2147/MDER.S241062] [Medline: 32104106]

39. Castillo AF, Davis AL, Fischhoff B, Krishnamurti T. Digital medicines for adherence support: a conceptual framework and qualitative study of adherence among chronically ill patients. Health Inf J. 2021;27(5):14604582211059463. [doi: 10.1177/14604582211059463] [Medline: 34825829]

29. Sarzynski E, Decker B, Thul A, et al. Beta testing a novel smartphone application to improve medication adherence. Telemed J E Health. Apr 2017;23(4):339-348. [doi: 10.1089/tmj.2016.0100] [Medline: 27564971]

59. Eggerth A, Hayn D, Schreier G. Medication management needs information and communications technology‐based approaches, including telehealth and artificial intelligence. Brit J Clin Pharmacol. Oct 2020;86(10):2000-2007. [doi: 10. 1111/bcp.14045]

46. Hincapie AL, Gupta V, Brown SA, Metzger AH. Exploring perceived barriers to medication adherence and the use of mobile technology in underserved patients with chronic conditions. J Pharm Pract. Apr 2019;32(2):147-153. [doi: 10. 1177/0897190017744953] [Medline: 29212410]

34. Faisal S, Ivo J, Tennant R, et al. Integration of a smart multidose blister package for medication intake: a mixed method ethnographic informed study of older adults with chronic diseases. PLoS One. 2022;17(1):e0262012. [doi: 10.1371/ journal.pone.0262012] [Medline: 35061773]

35. Shahani A, Nieva HR, Czado K, et al. An electronic pillbox intervention designed to improve medication safety during care transitions: challenges and lessons learned regarding implementation and evaluation. BMC Health Serv Res. Oct 30, 2022;22(1):1304. [doi: 10.1186/s12913-022-08702-y] [Medline: 36309744]

60. Hersberger KE, Boeni F, Arnet I. Dose-dispensing service as an intervention to improve adherence to polymedication. Expert Rev Clin Pharmacol. Jul 2013;6(4):413-421. [doi: 10.1586/17512433.2013.811829] [Medline: 23927669]

43. Anglada-Martínez H, Martin-Conde M, Rovira-Illamola M, et al. Feasibility and preliminary outcomes of a web and smartphone-based medication self-management platform for chronically Ill patients. J Med Syst. Apr 2016;40(4):99. [doi: 10.1007/s10916-016-0456-y] [Medline: 26872781]

61. Billingsley L, Carruth A. Use of technology to promote effective medication adherence. J Contin Educ Nurs. Aug 2015;46(8):340-342. [doi: 10.3928/00220124-20150721-12] [Medline: 26247653]

30. Brondi R, Bannò F, Bendinelli S, et al. Drugs don’t work in patients who don’t take them: Dr. Drin, the new ICT paradigm for chronic therapies. Stud Health Technol Inf. 2013;191:100-104. [Medline: 23792852]

93. Bakema R, Smirnova D, Biri D, Kocks JWH, Postma MJ, de Jong LA. The use of eHealth for pharmacotherapy management with patients with respiratory disease, cardiovascular disease, or diabetes: scoping review. J Med Internet Res. Sep 26, 2023;25:e42474. [doi: 10.2196/42474] [Medline: 37751232]

62. Thakkar J, Kurup R, Laba TL, et al. Mobile telephone text messaging for medication adherence in chronic disease: a meta-analysis. JAMA Intern Med. Mar 2016;176(3):340-349. [doi: 10.1001/jamainternmed.2015.7667] [Medline: 26831740]

88. Schneider MP, Gertsch A, Bugnon O. Cyberhealth serving to support individual intake of medication. Swiss Med Wkly. 2013;143:w13827. [doi: 10.4414/smw.2013.13827] [Medline: 23925717]

105. Tolley A, Hassan R, Grewal K, et al. Interventions to promote medication adherence for chronic diseases in India: a systematic review. Clin Med. Nov 2023;23(6):72-73. [doi: 10.7861/clinmed.23-6-s72]

94. Lammila-Escalera E, Greenfield G, Pan Z, Nicholls D, Majeed A, Hayhoe B. Interventions to improve medication adherence in adults with mental-physical multimorbidity in primary care: a systematic review. Br J Gen Pract. Jul 2024;74(744):e442-e448. [doi: 10.3399/BJGP.2023.0406] [Medline: 38429109]

63. Hugtenburg JG, Timmers L, Elders PJ, Vervloet M, van Dijk L. Definitions, variants, and causes of nonadherence with medication: a challenge for tailored interventions. Patient Preference Adherence. 2013;7:675-682. [doi: 10.2147/PPA. S29549] [Medline: 23874088]

64. Liu X, Varshney U. Mobile health: a carrot and stick intervention to improve medication adherence. Decis Support Syst. Jan 2020;128:113165. [doi: 10.1016/j.dss.2019.113165]

65. Marengoni A, Monaco A, Costa E, et al. Strategies to improve medication adherence in older persons: consensus statement from the Senior Italia Federanziani Advisory Board. Drugs Aging. Sep 2016;33(9):629-637. [doi: 10.1007/ s40266-016-0387-9] [Medline: 27444182]

66. Bingham JM, Black M, Anderson EJ, et al. Impact of telehealth interventions on medication adherence for patients with type 2 diabetes, hypertension, and/or dyslipidemia: a systematic review. Ann Pharmacother. May 2021;55(5):637-649. [doi: 10.1177/1060028020950726] [Medline: 32815400]

54. Wiecek E, Tonin FS, Torres-Robles A, Benrimoj SI, Fernandez-Llimos F, Garcia-Cardenas V. Temporal effectiveness of interventions to improve medication adherence: a network meta-analysis. PLoS One. 2019;14(3):e0213432. [doi: 10. 1371/journal.pone.0213432] [Medline: 30861014]

47. LaValley SA, Brady LA, Nguyen M, Jacobs D. Family caregivers of older adults: strategies, tools, and technologies for medication management. J Gerontol Soc Work. 2023;66(2):291-302. [doi: 10.1080/01634372.2022.2100856] [Medline: 35850634]

36. Fallah M, Yasini M. A medication reminder mobile app: does it work for different age ranges. Stud Health Technol Inf. 2017;235:68-72. [Medline: 28423757]

67. Kardas P, Bago M, Barnestein-Fonseca P, et al. Reimbursed medication adherence enhancing interventions in 12 European countries: current state of the art and future challenges. Front Pharmacol. 2022;13:944829. [doi: 10.3389/ fphar.2022.944829] [Medline: 36034792]

48. Donovan G, Hall N, Smith F, Ling J, Wilkes S. Two-way automated text messaging support from community pharmacies for medication taking in multiple long-term conditions: human-centered design with nominal group technique development study. JMIR Form Res. Dec 21, 2022;6(12):e41735. [doi: 10.2196/41735] [Medline: 36542458]

95. Doggrell SA. Adherence to medicines in the older-aged with chronic conditions: does intervention by an allied health professional help? Drugs Aging. Mar 1, 2010;27(3):239-254. [doi: 10.2165/11532870-000000000-00000] [Medline: 20210369]

41. Balasubramanian GV, Beaney P, Chambers R. Digital personal assistants are smart ways for assistive technology to aid the health and wellbeing of patients and carers. BMC Geriatr. Nov 15, 2021;21(1):643. [doi: 10.1186/s12877-021- 02436-y] [Medline: 34781881]

49. Backes C, Moyano C, Rimaud C, Bienvenu C, Schneider MP. Digital medication adherence support: could healthcare providers recommend mobile health apps? Front Med Technol. 2020;2:616242. [doi: 10.3389/fmedt.2020.616242] [Medline: 35047896]

98. Lancaster K, Abuzour A, Khaira M, et al. The use and effects of electronic health tools for patient self-monitoring and reporting of outcomes following medication use: systematic review. J Med Internet Res. Dec 18, 2018;20(12):e294. [doi: 10.2196/jmir.9284] [Medline: 30563822]

3. Hartch CE, Dietrich MS, Stolldorf DP. Effect of a medication adherence mobile phone app on medically underserved patients with chronic illness: preliminary efficacy study. JMIR Form Res. Dec 11, 2023;7:e50579. [doi: 10.2196/50579] [Medline: 38079192]

68. Torres-Robles A, Wiecek E, Tonin FS, Benrimoj SI, Fernandez-Llimos F, Garcia-Cardenas V. Comparison of interventions to improve long-term medication adherence across different clinical conditions: a systematic review with network meta-analysis. Front Pharmacol. 2018;9:1454. [doi: 10.3389/fphar.2018.01454] [Medline: 30618748]

69. Tantray J, Patel A, Wani SN, Kosey S, Prajapati BG. Prescription precision: a comprehensive review of intelligent prescription systems. Curr Pharm Des. 2024;30(34):2671-2684. [doi: 10.2174/0113816128321623240719104337] [Medline: 39092640]

102. Schneider MP, Krummenacher I, Figueiredo H, Marquis J, Bugnon O. Adherence: a review of education, research, practice and policy in Switzerland. Pharm Pract (Granada). Apr 2009;7(2):63-73. [doi: 10.4321/s1886- 36552009000200001] [Medline: 25152780]

70. Sun S, Simonsson O, McGarvey S, Torous J, Goldberg SB. Mobile phone interventions to improve health outcomes among patients with chronic diseases: an umbrella review and evidence synthesis from 34 meta-analyses. Lancet Digit Health. Nov 2024;6(11):e857-e870. [doi: 10.1016/S2589-7500(24)00119-5] [Medline: 39332937]

104. Yasmin F, Banu B, Zakir SM, Sauerborn R, Ali L, Souares A. Positive influence of short message service and voice call interventions on adherence and health outcomes in case of chronic disease care: a systematic review. BMC Med Inf Decis Making. Apr 22, 2016;16:46. [doi: 10.1186/s12911-016-0286-3] [Medline: 27106263]

71. Pirri S, Lorenzoni V, Turchetti G. Scoping review and bibliometric analysis of Big Data applications for medication adherence: an explorative methodological study to enhance consistency in literature. BMC Health Serv Res. Jul 24, 2020;20(1):688. [doi: 10.1186/s12913-020-05544-4] [Medline: 32709237]

44. Stecher C, Linnemayr S, Reaven P, Cloonan S, Huckfeldt P. Cost savings from an mHealth tool for improving medication adherence. Am J Managed Care. Oct 1, 2024;30(10):e289-e296. [doi: 10.37765/ajmc.2024.89621] [Medline: 39467173]

72. Schwartz JK, Grogan KA, Mutch MJ, et al. Intervention to improve medication management: qualitative outcomes from a phase I randomized controlled trial. Am J Occup Ther. 2017;71(6):7106240010p1-7106240010p10. [doi: 10.5014/ajot. 2017.021691] [Medline: 29135431]

73. Haugh KH. Medication adherence in older adults: the pillbox half full. Nurs Clin North Am. Jun 2014;49(2):183-199. [doi: 10.1016/j.cnur.2014.02.006] [Medline: 24846467]

74. Zullig LL, Ryan JS, Hayden BB. Applying technology to medication management and adherence. In: Marsch L, Lord S, Dallery J, editors. Behavioral Health Care and Technology: Using Science-Based Innovations to Transform Practice. Oxford University Press; 2014:81-94. [doi: 10.1093/med/9780199314027.003.0007]

75. Austin RR, Hull S. The power of mobile health technologies and prescribing apps. Comput Inf Nurs. Nov 2014;32(11):513-515. [doi: 10.1097/CIN.0000000000000120] [Medline: 25391038]

99. Aungst TD. Reevaluating medication adherence in the era of digital health. Expert Rev Med Devices. Dec 2021;18(sup1):25-35. [doi: 10.1080/17434440.2021.2019012] [Medline: 34913793]

96. Choi JY, Choi H, Seomun G, Kim EJ. Mobile-application-based interventions for patients with hypertension and ischemic heart disease: a systematic review. J Nurs Res. Oct 2020;28(5):e117. [doi: 10.1097/JNR.0000000000000382] [Medline: 32501962]

76. Viswanathan M, Golin CE, Jones CD, et al. Closing the quality gap: revisiting the state of the science (vol. 4: medication adherence interventions: comparative effectiveness). Evidence Rep Technol Assess (Full Rep). Sep 2012(208.4):1-685. [Medline: 24422970]

100. Mash R, Christian C, Chigwanda RV. Alternative mechanisms for delivery of medication in South Africa: a scoping review. S Afr Fam Pract. Aug 24, 2001;63(1):e1-e8. [doi: 10.4102/safp.v63i1.5274]

77. Aranda-Jan CB, Mohutsiwa-Dibe N, Loukanova S. Systematic review on what works, what does not work and why of implementation of mobile health (mHealth) projects in Africa. BMC Public Health. Feb 21, 2014;14:188. [doi: 10.1186/ 1471-2458-14-188] [Medline: 24555733]

97. Artificial intelligence in health care: benefits and challenges of technologies to augment patient care. US Government Accountability Office; Nov 30, 2020. URL: https://www.gao.gov/products/gao-21-7sp [Accessed 2026-08-11]78. Academy of Medical Sciences. Patient adherence to medicines. 2015. URL: https://acmedsci.ac.uk/policy/policyprojects/patient-adherence-to-medicines [Accessed 2025-09-05]

79. Khan R, Socha-Dietrich K. Investing in medication adherence improves health outcomes and health system efficiency. Organisation for Economic Co-operation and Development; 2018. URL: https://www.oecd.org/en/publications/ investing-in-medication-adherence-improves-health-outcomes-and-health-system-efficiency_8178962c-en.html [Accessed 2026-08-11]

80. HTA policy and methods review: emerging health technologies. Australian Government Department of Health and Aged Care; Jul 10, 2024. URL: https://www.health.gov.au/resources/publications/hta-policy-and-methods-review-emerginghealth-technologies?language=en [Accessed 2026-08-11]

103. Perry C, ChhatraliaK, Damesick D, Hobden S, Volpe L. Behavioural insights in health care: nudging to reduce inefficiency and waste. The Health Foundation; Dec 2015. URL: https://www.health.org.uk/reports-and-analysis/reports/ behavioural-insights-in-health-care [Accessed 2026-08-11]

81. Medication safety in polypharmacy: technical report. World Health Organization; 2019. URL: https://www.who.int/ publications/i/item/medication-safety-in-polypharmacy-technical-report [Accessed 2026-08-11]

82. Cross AJ, Elliott RA, Petrie K, Kuruvilla L, George J. Interventions for improving medication-taking ability and adherence in older adults prescribed multiple medications. Cochrane Database Syst Rev. May 8, 2020;5(5):CD012419. [doi: 10.1002/14651858.CD012419.pub2] [Medline: 32383493]

83. Nieuwlaat R, Wilczynski N, Navarro T, et al. Interventions for enhancing medication adherence. Cochrane Database Syst Rev. Nov 20, 2014;2014(11):CD000011. [doi: 10.1002/14651858.CD000011.pub4] [Medline: 25412402]

84. Coulter A, Ellins J. Patient-focused interventions: a review of the evidence. The Health Foundation; 2006. URL: http:// www.pickereurope.org/Filestore/Publications/QEI_Review_AB.pdf [Accessed 2025-09-05]

85. International Longevity Centre UK. Doctor’s Orders: Why adherence is critical to improving health outcomes across the life course. 2022. URL: https://ilcuk.org.uk/doctors-orders/ [Accessed 2025-09-05]

86. National Institute for Health and Care Excellence. YOURmeds for medication support in long-term conditions. 2023. URL: https://www.nice.org.uk/advice/mib289

106. Posadzki P, Mastellos N, Ryan R, et al. Automated telephone communication systems for preventive healthcare and management of long-term conditions. Cochrane Database Syst Rev. Dec 14, 2016;12(12):CD009921. [doi: 10.1002/ 14651858.CD009921.pub2] [Medline: 27960229]
