# Supplementary material for: Contexts and Mechanisms Related to the Efficacy of Digital Medication Adherence Interventions to Support Medication Adherence Among Adults With Chronic Diseases: Realist Review
Source: Interact J Med Res. 2026 Aug 28;15:e89803. doi: 10.2196/89803 (PMC13524365; doi:10.2196/89803)
Supplement: Supplementary file 4 [file ijmr-v15-e89803-s004.docx]

Summary of 35 CMOs in 7 theory areas with exemplar quotes to support the CMOs

| **Program theory** | **Source (Literature/Workshops/Combined)** | **Context** | **Mechanism** | **Outcome** | **Quotes to support CMOs from specific studies or workshops** |
| --- | --- | --- | --- | --- | --- |
| **Theory 1: Building and maintaining medication intake habits** | | | | | |
| CMO 1: Reminders for improving medication intake habits | Combined | Forgetfulness | 1. Receiving reminders to take medicines and keeping water ready to take with medicines that are customised in terms of content and frequency according to individual needs using text messages, mobile apps, or medication dispensers that provide sound and light reminders (mechanism resource) can reduce forgetfulness and strengthen habits of taking medications correctly at certain time (mechanism response). | 1. Improved confidence in taking medications independently  2. Improved medication adherence | *“One way to build a stronger habit is by reminding the patient when it is time to take their medication, such as with pill bottle caps that light up when it is time to take the medication. Another approach to strengthen a patient’s habit of taking medication is to leverage technology-based interventions and remind the patient to take their medication by sending a text message or alert on the patient’s phone when it is time to take their dose” (Badawy et al., 2020 [87])*  *“Maybe if there is an app alongside it that reminds you if you are out, that’s probably better”.* LM9 (logic model workshop 2)  *“I think the last time, one of the care recipients was saying that she sometimes gets the tablet out of the box, she wasn’t using this box, she made her own box. But she gets the tablet out of the box and because she doesn’t have a glass of water there ready, she goes into the kitchen and then takes quite a while and then forgets what she was doing and forgets that after the drink of water, she was getting her tablets. So, we talked about maybe having a jug or a glass of water ready next to it at all times.” (Facilitator1, Logic model workshop 2)* |
| CMO 2: Action plans for incorporating medication intake in daily routines | Combined | Forgetfulness | If an individual is using a smart medication device and the device provider monitors their medication adherence patterns and collaboratively makes action plans with them to incorporate medicine intake into daily routine activities like mealtimes or morning routines (mechanism resource), then it can strengthen habits of taking medicines at certain times (mechanism response) | Improved medication adherence | *“Cue-dose training entails reviewing pill cap data with patients and advising them to link medication-taking with an element of their daily routine such as brushing their teeth or mealtimes. Cue-dose training was associated with a statistically significant mean* *increase of 15% more metformin doses taken as prescribed compared to the control group.”* Zullig et al (2014) [74]  *“Habits are really important, medication adherence, linking it to brushing your teeth or whatever it is.”* LM9 (logic model workshop 2) |
| CMO 3: Re-establishing habits and routines related to medication intake after hospital discharge | Combined | Recent hospital discharge | Providing smart pillboxes/medication adherence devices with light and sound reminders, monitoring and feedback on medication intake and problem solving of barriers to medication adherence in case of many missed doses (mechanism resource) can help in easy access of medications and re-establishing their routines & habits related to medication adherence (mechanism response). | 1. Re-establishment of medication adherence.  2. Potential prevention of further deterioration of health and re-hospitalisation. | *“exit surveys with patients who used the pillbox were extremely positive. They often noted the convenience of their medications being mailed to them, increased confidence knowing it was the correct regimen, and no longer having the burden (or their family’s burden) to fill their own pillbox or use pill bottles to organize their regimens.”* Shahani et al., (2022) [35]  *“A pharmacist at the filling pharmacy also reviewed each patient’s medication adherence report on a weekly basis. In the event of > 20% missed doses for 3 days in a row, the pharmacist would call the patient to discuss possible barriers to medication adherence and create a plan to overcome these barriers.”* Shahani et al., (2022) [35]  *“That’s why it works so well with the hospital discharge is because if people are coming out of the hospital, we can monitor them using it” LM9 (Logic model workshop 2)*  “I *would say yeah, that’s really important isn’t it. Yeah, maintaining and maybe even improving independence after discharge.” LM2 (Logic model workshop 2)* |
| CMO 4: Low adoption due by patients after hospital discharge | Combined | Patients overwhelmed by recent hospitalisation and discharge from hospital | A smart medication device providing reminders to take medication, monitoring & feedback (mechanism resource) maybe overwhelming to the patient as they do not want additional changes to their routine after hospital discharge (mechanism response) | 1. Low adoption of the device.  2. Problems with building medication intake routines  3. Reduced medication adherence. | *“Other patients were simply overwhelmed by their hospitalization and did not want any additional changes to their routine.”* Shahani et al., (2022) [35]  *“People not wanting to be bothered straightaway when they get home from hospital. Being given the box just as people get home may be overwhelming. Risks are there if they decline support and have no one there to help them”*  *(Facilitator notes in Logic model workshop 2)* |
| CMO 5: Customising devices when travelling | Combined | Travelling | 1. Carrying a smaller smart medication box with features such as medication reminders and monitoring of adherence patterns (Mechanism resource) can reduce disruptions in the person’s medication routines during travel (mechanism response). However, this can cause some confusion regarding when to take their medications among some individuals if they pack only one strip of medicine to put in the smaller smart medication device while travelling as they would not have access to instructions for taking the medications that is often printed on the bigger medicine box (mechanism response).  2. Leaving the smart medication box at home and informing the device provider while receiving medication reminders from a mobile app (mechanism resource) can reduce disruptions in medication routines during travel (mechanism response).  3. Reminders for taking medications that can be customised to match the local time zone of travel (mechanism resource) can reduce disruptions in medication routines during travel (mechanism response) | Increased flexibility in routines. | *“Another one of the participants described struggles in remembering to take medications when traveling, particularly to places with a different time zone. As Susanna said: “If you’re travelling, you know, and you’re in [name of place] where the time changes so drastically...Should I take it at, what I would have taken in [name of place] or do I switch it?” Regarding this struggle, she noted that an SMRA feature that reminds of medication taking based on local time would be helpful as she continued:*  *Then the app would go off [to remind of medication taking].””* (Park et al., 2017 [40])  *“that’s something we could think about, varying box sizes with smaller boxes to make them more suitable.”* LM9 (Logic model workshop 2)  *“And I think an app alongside it is something that would be beneficial.”* LM9 (Logic model workshop 2) |
| CMO 6: Improving independence and flexibility | Workshops | A domiciliary care recipient wants more flexibility and independence in their daily routines | Using a smart medication device with features like reminders and monitoring and feedback on adherence and ongoing medication reviews to optimise the medication regimes (mechanism resource) can improve the individual’s confidence in taking medicines correctly leading to improved flexibility in their daily routines (mechanism response) | 1. Improved medication adherence  2. Improved health-related quality of life. | *“I think for those people who don’t want carers in their house, 2,3, 4 times a day who want to be independent, they want to go out, don’t want to be restricted with times, I think the independence that it can possibly give them and the control and autonomy over their medication regime, I think is a positive. I think what might not make it work as I’ve said before is if there is no ongoing support, meds review, following this device being put in and that maintenance ongoing I think is, umm, they would end up struggling again.”* (LM1, ICB staff involved in implementation of device, Logic model workshop 1) |
| CMO 7: Customising devices for individuals who enjoy spending time outdoors. | Workshops | Individual enjoys spending time outdoors, for example, in their garden | Linking the reminders provided by the smart medication devices installed in individuals’ homes to their phones, so that they can hear it through their earphones (mechanism resource) can ensure that they know when the reminders are being provided and it can improve individuals’ ability to take medicines when reminders are provided (mechanism response). | Improved habits of taking medicines at certain times and improved medication adherence. | *“Some people would be maybe like that alarm to go through to their phone if they were spending time in the garden or they've got their earphones or headphones and maybe it can be linked up with something like that.” (LM14, ICB pharmacist, logic model workshop 2)* |
| **Theory 2: Solving medication-related barriers to adherence.** | | | | | |
| CMO 8: Self-monitoring of medication adherence to reduce missed or double doses | Combined | Concerns for missed doses or double doses of medications | 1. Recording and self-monitoring of medication intake, for example, a mobile app which allows individuals to “check off” medicines (mechanism resource) they took so that they can document them can allow patients to have a reference for and see the medications they have taken already (mechanism response).  2. If the individual has been provided with a smart medication box or a mobile app which allows the individual to record their medication intake, the device providers can monitor the adherence patterns of the individual and prompt the individual to take their medications in case of missed doses (mechanism resource). This will help the individual to gain more knowledge and awareness about the correct timing of their medication doses (mechanism response). | 1. Reduced concerns for double doses or missed doses due to forgetfulness.  2. Increased independence in taking correct medications and improved medication adherence. | *“Participants wanted to “check off” the medicines as they took them in order to document which medicines they had taken. One participant suggested this feature could assist specifically with complex regimens if “you are taking seven medicines in the morning in a handful, you would want to be able to check them all off at once.” This feature could serve as a reference for which medicines they took and could alleviate their concerns about missed doses or accidental double dosing.” Russell et al., (2018) [45]*  *“One example of how AI might assist in improving adherence is in the case of direct oral anticoagulants in which an AI system embedded in smartphones was used to directly observe patients taking the medications. The AI incorporated imaging systems such as facial recognition and medication identification as well as analytics to identify those at high risk or to confirm delay in administration. Those who were identified as being at high risk of non-adherence were routed to a study team for in-person outreach as needed. In a 12-week randomized controlled trial format, the AI arm had 100 percent adherence and the control arm had 50 percent adherence by plasma drug concentration level assessment.”* (United States Government Accountability Office  & national academy of medicine, 2022 [97])  *“I do think that's a really smart feature of it that it's process driven. So, if as you say someone forgets or they're not, you know, they're not responding to it in time, it keeps escalating to the point then there's an intervention from you to check on them. Sometimes that's been the case, especially with my dad's because he's on strong blood thinners and there is a window that he has to have it. So, if you don't check in with him, it can have a detrimental effect. So, I think that that's a good feature of the box that you were just talking about.”* LM3 (Logic model workshop 2) |
| CMO 9: Helping individuals to cope with changes in medication regimes | Combined | Changes in medication regimes | 1. Recording names or images of medications in a mobile app or including a virtual pillbox with an option to add shape and colour of medications and audibly announcing medication names and showing their pictures while providing reminders (mechanism resource), enable better recognition and learning of the medicines (mechanism response).  2. If an individual is using a smart medication device, collaboration between the device provider and user to update the contents of device when medication regimes change (mechanism resource) can increase the knowledge of the changed medication regime among the device provider and the user (mechanism response). | Improved adherence to the changed medication regime. | *“In addition, Emily described an SMRA feature that enables recording medications in the app in visually precise ways (ie, virtual pill box) as helpful for managing varying medications, as she said:*  *“I did like how that had, um, the pills you could put the colour and the shape...I thought that was nice. Especially because they do change so often.””* (Park et al., 2017 [40]).  *“People should have capacity to inform device provider when their medication regimes change and the device provider will review box contents at 3 and 6 weeks” (Facilitator notes in Logic model workshop 1)* |
| CMO 10: Helping individuals cope with temporary medications | Combined | Individual prescribed with temporary medications | 1. If an individual is using a smart medication device, temporary medications should be kept inside the device by the user, and the device provider should be informed about the prescription of the temporary medications (mechanism resource) which will ensure that the device provider is cognizant of the prescription of temporary medications (mechanism response). The device provider can remotely reset the box after the temporary medications are no longer required (mechanism resource).  2. If an individual is using a smart medication device, temporary medications could be kept outside the box and text message reminders to take these medicines could be set up or paper notes with the names of these medications could be included inside the device (mechanism resource). This will act as a reminder for the individual to take the temporary medications and increase awareness about them (mechanism response). | 1. Improved medication adherence  2. Improved health-related quality of life in the long-term. | *“Yeah, I was saying that the temporary medications could be remotely updated on the system” LM13 (Logic model workshop 2)*  *“So, if they are, let’s say they have antibiotics and they put them in the box and we can see that they are opening the box more often that week, that would trigger us to speak to that person.” LM9 (Logic model workshop 2)*  *“Too many medications dispensed outside of the pillbox: Text reminders for non-pillbox medications, adopted with moderate success”* Shahani et al., (2022) [35].  *“frequently changing medications (e.g., diuretics, warfarin), as-needed (PRN) medications, and opioids and other controlled substances were not included in the medication trays.”* Shahani et al., (2022) [35]. |
| CMO 11: Improving medication adherence among individuals prescribed with PRN medications | Combined | Individuals prescribed with PRN or “when required” or “as needed medications” | If an individual is using a smart medication device such as a smart medication box to take their medications, the PRN medications should be left out of the device and notes with the names of the PRN medications should be included in the box or text message reminders could be created as reminders for PRN medications (mechanism resource). This will act as a reminder for the individual and increase awareness about the PRN medications if they are required (mechanism response). | 1. PRN medications do not interfere with the monitoring of adherence of other medications.  2. Accurate medication adherence monitoring. | *“Too many medications dispensed outside of the pillbox: Text reminders for non-pillbox medications, adopted with moderate success”* Shahani et al., (2022) [35].  *“Frequently changing medications (e.g., diuretics, warfarin), as-needed (PRN) medications, and opioids and other controlled substances were not included in the medication trays.”* Shahani et al., (2022) [35].  *“He has a note inside his box as well and to say, you know, have you know, have you checked your (name of PRN medication) and you know having other things in place as well because PRNs it won't necessarily be the same reminders at the same times, it won't be prompted in the same way” LM9 (Logic model workshop 2)* |
| CMO 12: Helping individuals cope with complex medication regimes | Workshops | Individual prescribed with complex medication regimes | 1. If an individual is using a smart medication device (eg, a smart medication box) to take their medications, a chart with name and the schedule to take each medication inside the device (mechanism resource) can improve the individual’s knowledge of their medications (mechanism response).  2. If the provider of the smart medication device arranges any support for the individual if they require it such as recommending the individual to schedule an appointment with the GP or clinical pharmacists to optimise their medications (mechanism resource), then it can improve the individual’s understanding of arranging a medication review to simplify their medication regimen (mechanism response). | 1. Improved medication adherence.  2. Improved health-related quality of life in the long-term. | *“What we try to do in the start is that see the support they already have in place. So, do they need referring to the clinical pharmacist or the surgery? Do the medication support team need to be involved? Does some of it need changing? We will help with that, we will signpost that and manage that meeting has taken place. Speak to the surgery to sort of try and optimise their medication before it goes into here (in the box).”* LM9 (Logic model workshop 2).  *“Option to have a chart in the box to say what medicine to take when” (Facilitator notes in Logic model workshop 1)* |
| **Theory 3: Enabling collection of prescription medications on time.** | | | | | |
| CMO 13: Enabling easier refill of prescriptions | Literature | Individual facing barriers like difficulty renewing prescriptions or reordering prescriptions | 1. Providing text messages to patients outlining the process of ordering medication from a prescriber and collecting it from a pharmacy (mechanism resource) can improve understanding on how to perform these behaviours (mechanism response).  2. Electronic reminders for refills (mechanism resource) can help patients remember to refill their prescriptions in advance (mechanism response).  3.Providing longer prescriptions (mechanism resource) can reduce patients’ burden of repeatedly re-ordering prescriptions (mechanism response) | Improved medication adherence due to easier access to medications. | *“Performing the behavior obtaining medication is a pre‐requisite to the behavior taking medication. Without physical opportunity, taking medication is unlikely to occur. Behaviour Change Techniques were identified as targeting obtaining medication where interventions influenced activities such as ordering medication from a prescriber and/or collecting it from a pharmacy. The most common BCT used to target this behavior was Instruction on how to perform a behavior using automated digital communication. Interventions that included BCTs to target obtaining medication had an almost universally positive impact on medication adherence with 90% of studies targeting this behavior finding improvements. This suggests that this behavior is a good target where physical opportunity is a barrier to taking medication.”* Donovan et al., (2022) [48]  *“Initiatives like long-term medication supply, online refills of prescriptions, mail order pharmacy use, and calendarised blister* *packaging all have been shown to significantly improve adherence (Schmittdiel, et al., 2015; Zedler et al., 2011).”* Khan and Socha-Dietrich (2018) [79]  *“Electronic systems may also be able to notify a prescriber or pharmacist about refills, which can help trigger an intervention to avoid a potential gap in medication use.”* Khan and Socha-Dietrich (2018) [79] |
| CMO 14: Enabling easier access to prescription medicines in LMICs | Literature | LMICs with lack of infrastructure and human resources where refilling prescriptions are difficult due to long waiting times | Installing pharmacy dispensing units which allows remote dispensing of medications (dispensing can be requested online) in convenient locations for patients to collect later and/or smart lockers in accessible locations which store prescribed medications for patients to collect with a password (mechanism resource) can make it more convenient for individuals to collect prescriptions (mechanism response). There must be an electronic communication system to inform patients on date of delivery and venue, and a reliable record should be there to monitor the safety, status and location of medication packages (mechanism resource). | Improved medication adherence due to easier access to medications. | *“The primary health care services are struggling to cope with the increasing patient numbers and are themselves often less than ideal, with poor infrastructure, limited human resources, unreliable supply chains and paper-based health information systems. Patients experience long waiting times to obtain medication, have to miss work and lose income, while facilities are often congested by patients that are stable and only need medication. This unacceptable experience may lead patients to avoid collecting their medication, which contributes to poor adherence and control of chronic diseases.”* Mash et al (2001) [100].  *“New technology such as smart lockers and automated pharmacy dispensing units have been piloted. Patients benefited from these alternatives and had improved adherence.”* Mash et al (2001) [100]. |
| **Theory 4: Supporting people with sensory or motor impairments and unpredictable health issues** | | | | | |
| CMO 15: Effective telecare monitoring | Combined | Individuals with unpredictable health conditions that can lead to dizziness and falls. | 1.Using electronic smart medication devices, dispensers, pill boxes or blister packs which can be used by healthcare professionals or device providers to monitor and provide feedback on missed doses, adherence patterns and health statuses (mechanism resource) can help the individual gain more awareness of their adherence patterns (mechanism response).  2. Monitoring of health status and informing informal carers and emergency in case of a health deterioration or a fall (mechanism resource) can lead to increased feelings of safety and reduced anxiety of future falls (mechanism response). | 1. Reduced chances of missed doses.  2. Effective telecare home monitoring in case of health deterioration due to low adherence.  3. Effective intervention in case of dizziness and falls. | *“The ODP-MSS works as a medication dispenser, simultaneously encapsulating individual doses of multiple medications in separate film pouches. The system can use music alerts to remind patients to take their medication. If the patient misses a dose, a voice message is sent by telephone to a medication reminder. The system also allows the nurse to check the patient’s health status over the phone. Patients using the ODP-MSS can press a “going-out” button to pre-dose the next ODP before they leave. The ODP-MSS has an internal memory to store medication-related data, such as medication removal status, automatic call events, and going-out switch activity. This internal memory enables the assessment of medication adherence. The researchers found that the device effectively prevented patients from missing doses and provided telecare home monitoring for older people living independently at home.”* (Gackowski et al., 2024 [52]) |
| CMO 16: Adapting devices for adults with dexterity problems | Combined | 1. Older adults with dexterity problems.  2. Young children often visit the older adult with dexterity problems | 1. Using electronic pillboxes or dispensers with bigger compartments/pill trays/drawers in which medications are stored securely (mechanism resource) can enable easier access to medications (mechanism response).  2. If young children visit the older adult often, the smart medication devices provided to them may need to be locked (mechanism resource) which can cause problems with accessing medicines if the individual has dexterity problems (mechanism response) | 1. Improved medication adherence if electronic pillboxes are adapted to the dexterity levels of older adults.  2. Poor medication adherence if the device needs to be locked to prevent interference by young children. | *“As older adults in some cases might have arthritis, taking the medication out of the dispensing pill tray/opening/drawer could be difficult for them. Therefore, special care should be taken when designing the size/ capacity of the pill tray/opening/drawer; they should be adequate, and the medication must be secure and not become lost/fall out.”* (Miguel-Cruz et al, 2019 [56])  *“Eligibility criteria is they should have dexterity to open and close the box.” (Facilitator notes in Logic model workshop 1)*  *“Umm, just to kind of add on to the dexterity side of stuff, obviously that’s part of the eligibility criteria but umm, sometimes there is no coordination of movements as well, umm, which might be tricky for individuals to actually manage to do independently.” LM13 (Logic model workshop 2)* |
| CMO 17: Customising mobile apps for people with visual impairment | Literature | Individuals with visual impairment | Adapting mobile apps to improve medication adherence to have big text sizes and appropriate background colours (mechanism resource) can improve perceived ease of use (mechanism response). | Increased intention to use the technology to improve medication adherence. | *“According to the 23 patients and 7 health professionals that participated in the qualitative research process, the design of the ALICE app had to satisfy the following criteria: it needed to be easy to use, have sufficiently large font size, include photographs of the medications.”* (Mira et al., 2014 [32])  *“Results revealed that the level of perceived ease of use and the level of intention to use an SMRA were higher in the training group than in the nontraining group, and the focus groups indicated that perceived ease of use might lead to intention to use the app.”* (Park et al., 2017 [40])  *“These findings expand existing studies on middle-aged to older patients’ Smartphone Medication Reminder App use by indicating not only the utility of a training session in promoting patients’ app use but the type and focus of a training session that could be a promising intervention to promote patients’ app use: a scheduled small-group training session in a hospital setting focused on helping patients feel at ease navigating and using the app by providing them with step-by-step instructions on and hands-on experience with app features.”*  (Park et al., 2017 [40]). |
| CMO 18: Devices that are suitable for individuals with visual impairments | Literature | People with visual impairments | Assistive devices like Alexa that can provide reminders to take medications at certain times, and features like voice commands which can be used to ask questions about medications and health conditions (mechanism resource) can reduce forgetfulness and improve knowledge of medications (mechanism response). | Improved medication adherence | *“Moreover, patients could ask the device medical questions and side-effects of drugs and feel better informed. Users with visual impairment benefitted from the voice-based features the most.”* (Balasubramanian et al, 2021 [41]) |
| CMO 19: Adapting devices for individuals with hearing impairment | Workshops | Individuals with hearing impairment | Using a smart medication device that providers light in addition to sound medication reminders installed in a location that is accessible for the individual (mechanism resource) can help them to be cognizant of the reminders to take medications (mechanism response). | 1. Stronger habits of taking medications at certain times.  2. Improved medication adherence. | *“Problem with hearing, so doesn’t know the buzzer is going off-discussion with (Company name) about location of box in an accessible place in the person’s home” (Facilitator notes in Logic model workshop 1)* |
| **Theory 5: Supporting individuals with high anxiety, low medication self-efficacy and low social support** | | | | | |
| CMO 20: Preventing social isolation due to reduction in care packages. | Workshops | Domiciliary care recipient lives alone and enjoys care providers company when they visit. | Assessing the potential for social isolation and deterioration of mental health after giving a smart medication device to the individual and providing appropriate social support in case of social isolation (mechanism resource) can help the individual feel supported (mechanism response). | Prevention of deterioration of mental health | *“I think the area that for some people may not be helpful is if they are very isolated and they see these visits as someone to chat with and that is then taken away from them, that would then increase their social isolation. So, that would be the downside of it I think.” LM5 (Logic model workshop 1)* |
| CMO 21: Supporting individuals with high anxiety and concerns about managing their medications independently when smart medication device is taken away. | Workshops | 1. Concerns about the individual’s ability to manage their medications after using the smart medication device for 6-12 weeks when the device will be taken away.  2. Anxiety of the device being taken away after developing medication intake habits for 6-12 weeks. | 1. Suggestions from the device provider to the domiciliary care recipient and informal and formal care providers regarding alternative ways to manage medications when the device is being taken away (mechanism resource) can help the individual to try out and become comfortable with different technologies to take their medications (mechanism response).  2. If the individual can keep the smart medication box but not the reminder attached to it (mechanism resource), they will develop stronger habits of taking the medicines out of the same place (mechanism response). However, the fact that the device was being taken away after they use it for some time might cause some individuals to refuse to use it (mechanism response). | 1. Reduced anxiety and good medication adherence    2. Low adoption of the device among some individuals as it is being taken away after 6-12 weeks which can affect scalability if many individuals refuse to use it. | *“End of the project: keep the box but not the reminder device. Helps storing medicines in one place. Keeps routine intact” Facilitator notes from Logic model workshop 1).*  *“So, they keep the box, but they don’t have the little smart device. So, the idea is that they build their independence.” LM2 (Logic model workshop 2)*  *So, at the end of that period, if they didn’t feel comfortable, we would not just walk away with that information. So, having a good offboarding is really important. It could be that they could just have an app or something, depending on how that person is. LM9 (Logic model workshop 2)*  *“If you take the box away from them, if they become reliant on it or if they don’t quite understand how to use it the first time you have shown them and then they get anxiety from that.” LM3 (Logic model workshop 1).* |
| **Theory 6: Supporting individuals who are uncomfortable with technology or certain aspects of technology** | | | | | |
| CMO 22: Increasing perceived usefulness and acceptability of technology for older adults. | Combined | For individuals who are not comfortable with technology | 1. Providing individuals with some training on how to use the smart medication technology (mechanism resource) can help perceived ease of use of the device (mechanism response)  2. Highlighting the real-world applications of the technology in situations like emergencies and highlighting how their family members or doctors may perceive the features of the technology as useful to help improve their medication adherence (mechanism resource) can help in improving the subjective norm and perceived usefulness of the technology (mechanism response). | Increased intention to use the technology to take their medicines correctly. | *“Some participants described that family members could find patients’ SMRA use to be positive. Emily said: “For loved ones too, if anything ever happens to me, they could take my phone...if I end up in the emergency room...they’ll know everything that I take, will be right there on my phone.””* Park et al., (2017) [40]  *“Participants indicated that family members or health care providers might view participants’ SMRA use more positively because they perceive it to be useful in helping participants stay healthy, both in a day-to-day sense, as well as during an emergency. In sum, it was indicated that introducing participants to the utility of SMRA in medication adherence without applying it to the real-world setting might lead the intervention training session to not adequately target perceived usefulness and positive subjective norm.”* Park et al., (2017) [40]  *“Support and training to use the system: initial installation and ongoing” LM 5 (Facilitator notes in Logic model workshop 1)*  *“Care recipient who is uncertain about technology: Reassurance about the simple design of device” LM 9 (Facilitator notes in Logic model workshop 1)* |
| CMO 23: Adapting devices/interventions for informal carers of older adults | Combined | Individuals living with informal carers to help them with their medications | 1. Providing tailored information and training to the informal carers regarding the how to use the smart medication device (mechanism resource) can ensure the older adult receives better support to use the device to take their medication correctly (mechanism response)  2. If a smart medication dispenser has been provided, allowing early dispensing of medications or having an option like “user not at home” when the older adult is not at home or busy and allowing later dispensing of medications (mechanism resource), can prevent informal carers to take the medicines out of the smart dispenser on the behalf of the patient or silence the reminders from the device (mechanism response). | 1. Enabling accurate monitoring of medication adherence.  2. Improved medication adherence. | *“A few of the participants living with a spouse or a family member reported that their medications were dispensed by someone else to silence the alerts. The MDS assumed 100% adherence as soon as medications were dispensed. The manufacturers of the MDS can potentially enhance the safety and measurement of the system by only dispensing medications based on the finger-print scan of the registered user. In order to silence the system, there should be an option on the touch screen, such as “user not home”, where reminders would be delayed for one or 2 hrs based on the second prompted question “when will the registered user be home?””* Ahmad et al (2020) [43]  *“Issues about risks when family is supporting. Risks of family “interfering” with the box-information and training for family carers” (Facilitator notes based on discussion in Logic model workshop1).* |
| CMO 24: Adapting interventions for low resource conditions | Literature | Individuals of lower socio-economic status and/or individuals living in areas with limited healthcare facing barriers such as low technological literacy, limited access to mobile phones and limited internet access | Using a mix of non-technological and technological solutions to manage medications or simple devices that are easy to understand, use and install (mechanism resource) can help in improving accessibility to medication adherence interventions  (mechanism response). | Improved medication adherence and health-related quality of life | *“Less than one-third of patients had smartphones and most had limited access to the computer or Internet. Thus, there are mixed results on the feasibility of adopting mHealth methods to improve medication adherence. For some patients, various features of mHealth technology seem beneficial, but lack of understanding and complexity of utilizing these features, potential lack of resources, or unwillingness to pay for it may impact its use. These results are consistent with previous research related to the use of mobile technology in underserved populations.”* Hincapie et al., (2019) [46]  *“This research demonstrated that developing and promoting multiple strategies that encompass both technological and nontechnological-based approaches might be more effective for underserved patients.”* Hincapie et al., (2019) [46]  *“Simple to use, no wi-fi needed because of SIM card”* (*Facilitator notes based on discussion in Logic model workshop1)* |
| CMO 25: Customising interventions for the needs of informal carers and older adults. | Literature | Informal carers and older adults have non-technological systems in place to manage their medications and are uncomfortable with using technology | Using non-technological solutions like manual pillboxes or using the established systems they have to manage their medications (mechanism resource) will lead to lower caregiver burden and ensure that older adults and caregivers are comfortable with collaboratively managing the older adults’ medication intake (mechanism response). | Improved medication adherence and quality of life among older adults | *“In a technologically evolving landscape, it is critical to recognize that not all caregivers and their loved ones will benefit by incorporating new tools or strategies into their already busy caregiving situations, but may find traditional, non-technologically oriented strategies (i.e., manual pillboxes, plastic bags for medication storage or handwritten lists) effective for medication management.”* LaValley et al., (2023) [47] |
| CMO 26: automated technologies like IVR | Literature | 1. Individual prefers speaking to a person rather than an automated technology.  2. Individual requiring immediate support with medications | Receiving reminders to take medications or refill prescriptions through automated telephone technologies like interactive voice recognition systems (mechanism resource) may lead to feelings of frustration (Mechanism response). | Reduced acceptability of device | *“many participants were not receptive to the IVR technology. One reason was a frustration with not being able to talk to a real person: "I was irritated because I could not really speak to anybody and/or ask questions", "sometimes the calls were annoying and talking to a machine was not helpful", and "a machine is a machine. There is nothing human about that. I'd rather talk to a real person."* Reidel et al., (2008) [38]  *“ATCS may also present difficulties with the provision of immediate participant support.* *Others may simply have a strong preference for interactions with humans rather than with automated voice messages.”* Posadzki et al., (2016) [106] |
| CMO 27: “Alert fatigue” due to repeated reminder sounds from smart medication devices. | Workshops | A domiciliary care recipient is annoyed by repeated sounds | If an individual is annoyed by repeated sounds, using a smart medication device which provide repeated sound medication reminders (mechanism resource) can lead to the recipient shutting the smart medication device without taking the medicine or taking the wrong medicine to stop the sounds (mechanism response). This could be solved by customising the reminders and by allowing the device to emit the sound only once while providing reminders (mechanism resource) leading to improved comfort with using the device (mechanism response). | 1. Improved medication adherence.  2. Improved health-related quality of life | *“Taking the wrong medication if person is annoyed or just opens it to turn the alarm off” (Facilitator notes in Logic model workshop 1)* |
| **Theory 7: Economic, policy and organisational factors affecting implementation of smart medication devices** | | | | | |
| CMO 28: Problems in healthcare systems affecting provision of smart medication device before hospital discharge. | literature | Rushed nature of hospital discharge process and poor coordination and communication among different professionals during discharge | Preparation of the smart medication device like a pillbox on time and educating the patient about how to use it before discharge (mechanism response) can be hampered if the clinicians do not provide the prescriptions to the pharmacist on time (mechanism resource) | Low adoption of the device because of problems with implementation | *“Lack of compatibility between the rushed and unpredictable nature of hospital discharge with the time required to prepare the pillbox”* Shahani et al., (2022) [35]  *“So the so there's this kind of need, so if the idea is that people who are discharged from hospital get discharged with a Medy box, that could do with being written into the discharge letter so that the community pharmacy know and anybody's looking after the patient knows because those letters goes to the patient as well as well as the GP.” (LM14, ICB pharmacist, logic model workshop 2).* |
| CMO 29: Contexts associated with low motivation among care providers to refer recipients to use devices. | Workshops | 1. Domiciliary care providers worried about the reduced need for care and therefore reduced revenue for them.  2. Care provision is commissioned with rigid specifications of time and tasks, and domiciliary care providers have to complete a lot of administrative tasks. There are frequent gaps in the care visits that are commissioned with early morning and late-night care visits being common. Therefore a few medication prompting care visits during the day would increase the hours in their work shifts.  3. Care providers are risk averse and feel that they would be accountable to Care Quality Commission in case the care recipient they care for miss doses of medicines. | Care providers may not be motivated to refer care recipients (mechanism response) to use a smart medication device with features of medication reminders and monitoring and feedback on medication adherence (mechanism response) | 1. Reduced referrals of care recipients to use the device.  2. Reduced scalability of the intervention. | *“I just think that they have no real drive, unless the care providers are really caring, and they genuinely want to improve independence which some, a couple do. Then you are battling against their revenue. I really think that’s the main driver. I mean why would they want to do it. If they are not, there’s no real kind of, obviously it helps people’s independence but if that’s not enough of a driver then there’s really no reason for them to participate because it is added admin work for them.*  *LM9 (Logic model workshop 2)*  *“There are huge challenges there in social care and that has been, it has been for some time and then the work itself in terms of particularly domiciliary care and how it's commissioned. So, you have got a visit in the morning and a visit at lunch time and you have got a visit at teatime and a visit at night, and you have got gaps during the day. So, you know, there’s lots of challenges, so like there’s gaps during the day and you are not paid for those gaps. So, there are lots of challenges. I know from some providers I work with, that there are more providers in the market now in Lancashire. I know there are some providers saying that they are not getting enough referrals through, enough work through. So, there’s a lot happening in the dynamics in the market I think, so I think they are having an impact in a not so good way.” LM2 (Logic model workshop 2)*  *“Everyone’s very risk averse, you know. With the care providers, it was like “Do we need to report it to CQC if they miss it? Like what’s the criteria here?” So, the way that that works is that, during the first period, they are checking it, and they are responsible. They are responsible, however, that person has the capacity to show and will be self-medicating in that second period with the support of the device yes but if they miss a medication, that’s not a CQC report because they are self-medicating during that time and that has been done based on the data from the first few weeks and feedback.” LM9 (Logic model workshop 2)* |
| CMO 30: Financial incentives to increase referrals of recipients to use the device. | Workshops | Domiciliary care providers are unmotivated to refer care recipients to use the smart medication device being implemented in the community | Small financial incentives (£25-30) for each referral of a care recipient to use the smart medication device (mechanism resource) may not increase care providers’ motivation to refer more care recipients to use the smart medication device due to long-term reduction in revenue for them (mechanism response) | 1. Limited referrals of care recipients to use the device.  2. Low scalability of the intervention.  3. Limited cost savings due to reallocation of costs. | *“I think that the problem is, yeah, we are offering incentives like around 25-30 pounds to help cover the cost, but they are losing long-term care hours, so there’s not going to be any cost savings from this if we are incentivising this too much, it’s just reallocating the cost.” LM9 (Logic model workshop 2)* |
| CMO 31: Making use of smart medication devices mandatory when care recipient needs medication support. | Workshops | Domiciliary care providers are unmotivated to refer care recipients to use the smart medication device being implemented in the community | Local council policies to make the use of a smart medication device mandatory or a report outlining the reasons why a care recipient is not suitable for the use of a smart medication device to take their medications (mechanism resource) can improve care providers’ motivation to refer care recipients to use the smart medication device (mechanism response). | 1. More frequent referrals of domiciliary care recipients to use the device.  2. Better and more targeted care provision.  3. Potentially increased cost savings in care provision. | *“Yeah that’s, only way is to mandate it. So, like our organisation and another council, that’s what they have done, it’s the council who have done it, council authorities. So, every time a new care package is brought to them, they have to have a report to back up why the technology isn’t suitable or use the technology to inform that decision. If it doesn’t have that report with it, it gets thrown back at them.” LM9 (Logic model workshop 2)* |
| CMO 32: Digitization of social care | Workshops | Domiciliary care providers uncertain about how digitization of social care needs to be implemented by them in practice. | If support is provided to domiciliary care providers to implement technologies like smart medication devices in practice and healthcare systems supports collaborations between domiciliary care providers and technology providers (mechanism resource), then care providers will feel more confident in supporting care recipients to use such technologies (mechanism response). | 1. More frequent referrals of domiciliary care recipients to use the device.  2. Better and more targeted care provision. | *“I mean it is very much a strategic decision, and they are looking at care digitisation and we don’t know what it actually looks like in practice. So, there’s a huge confidence thing around that as well isn’t it. So, I think some providers are going to need a bit of a hand holding through that process as well. that’s interesting that how it came across to me that care provider and the tech provider working together and that really is what we should be looking at.” LM2 (Logic model workshop 2)* |
| CMO 33: Anxiety regarding reduced disability benefits hampering implementation of devices. | Workshops | Domiciliary care recipients worried about their disability benefits being reduced because of reduced need for care. | Individual may not be motivated (mechanism response) to use a smart medication device with features of medication reminders and monitoring and feedback on medication adherence (mechanism response) | Reduced adoption of the device. | *“Yeah, but I think their fear may be that if I use this tool, then my care will change or get reduced and as a result I may, or they may end up also losing their umm, I think its called independence payment, right? And and combined, that can be a reason for them to resisting to using the technology.” LM 12 (Logic model workshop 1)* |
| CMO 34: Instability in health and social care sector as a barrier to implementation of devices. | Workshops | Instability in health and care sector because of care home closures & staff storages in pandemic, more care providers available than required now and plans to implement cost savings in care sector. | Implementing smart medication devices in the community and providing them to domiciliary care recipients and patients who have recently been discharged from hospital (mechanism resource) will lead to feelings of frustration and reduced motivation among those involved in implementing the device and referring individuals to use the device (mechanism response). This can be mitigated in the future by allowing adequate time to assess the wider contexts in which the device is being implemented and form effective work pathways for implementing it (mechanism resource) which can improve learning regarding effective ways of implementing similar devices among professionals involved in implementation of similar devices (mechanism response). | 1. Limited scalability of the intervention.  2. Limited referrals and cost savings in care provision. | *“Re-structuring of all healthcare, LCC re-did framework last year, social workers now to introduce technology-this is a new policy, number of domiciliary care providers opening and closing during pandemic” (Facilitator notes in Logic model workshop 2)*  *“And what if it is freeing capacity for the care providers because they are drawing up the staff to meet the demand.” LM2 (Logic model workshop 2)*  *“Yeah, I think perhaps that situation was more like 18 months ago, but I think what the framework now is, it has opened up to a lot more, a lot less in that position.” LM 9 (Logic model workshop 2)*  *“I mean to embed anything, especially into health and social takes time, years like LM2 said years whereas now it’s like we are up against this target, we are 6 months in and we have spoken to all these teams, we have spoken to 28 providers. Now we should probably start looking at, right now let’s start looking at devices going in. But there was no time that was allocated once the provider was in place to develop those pathways, the service design piece.” LM9 (Logic model workshop 2)* |
| CMO 35: Creating awareness of devices among professionals assessing care needs to improve implementation | Workshops | Social workers and occupational therapists often assess the needs in terms of care provision of individuals | Creating awareness of the smart medication device, explaining its benefits and providing training on how to use it to social workers, occupational therapists and higher authorities (mechanism resource) may help in increased awareness of the benefits of the device among professionals who can help in referring individuals to use the device (mechanism response). | Increased referrals of appropriate care recipients or individuals recently discharged from hospital to use the device. | *“So, within actual social worker teams, social workers and occupational therapists are actually going out doing assessments with the individual and them having the awareness that something like this is available is important. It would be good to directly work with them and do something like a lunch at a team meeting that they have and then have a bit of lunch and demonstrate and explain stuff like that. But umm, obviously you have got the hospital discharge side of things as well so a lot of the times you have the hospital social workers or the therapists that are recommending what someone is going to need on discharge, umm, from a care perspective and whether something like that can be implemented there for each person as well.” LM13 (Logic model workshop 2)* |

**References**

87. Badawy SM, Shah R, Beg U, Heneghan MB. Habit strength, medication adherence, and habit-based mobile health interventions across chronic medical conditions: systematic review. J Med Internet Res. Apr 28, 2020;22(4):e17883. [doi: 10.2196/17883] [Medline: 32343250]

74. Zullig LL, Ryan JS, Hayden BB. Applying technology to medication management and adherence. In: Marsch L, Lord S, Dallery J, editors. Behavioral Health Care and Technology: Using Science-Based Innovations to Transform Practice. Oxford University Press; 2014:81-94. [doi: 10.1093/med/9780199314027.003.0007]

35. Shahani A, Nieva HR, Czado K, et al. An electronic pillbox intervention designed to improve medication safety during care transitions: challenges and lessons learned regarding implementation and evaluation. BMC Health Serv Res. Oct 30, 2022;22(1):1304. [doi: 10.1186/s12913-022-08702-y] [Medline: 36309744]

40. Park DY, Goering EM, Head KJ, Bartlett Ellis RJ. Implications for training on smartphone medication reminder app use by adults with chronic conditions: pilot study applying the technology acceptance model. JMIR Form Res. Nov 10, 2017;1(1):e5. [doi: 10.2196/formative.8027] [Medline: 30684397]

45. Russell AM, Smith SG, Bailey SC, et al. Older adult preferences of mobile application functionality supporting medication self-management. J Health Commun. 2018;23(12):1064-1071. [doi: 10.1080/10810730.2018.1554728] [Medline: 30526400]

97. Artificial intelligence in health care: benefits and challenges of technologies to augment patient care. US Government Accountability Office; Nov 30, 2020. URL: <https://www.gao.gov/products/gao-21-7sp> [Accessed 2026-08-11]

48. Donovan G, Hall N, Smith F, Ling J, Wilkes S. Two-way automated text messaging support from community pharmacies for medication taking in multiple long-term conditions: human-centered design with nominal group technique development study. JMIR Form Res. Dec 21, 2022;6(12):e41735. [doi: 10.2196/41735] [Medline: 36542458]

79. Khan R, Socha-Dietrich K. Investing in medication adherence improves health outcomes and health system efficiency. Organisation for Economic Co-operation and Development; 2018. URL: https://www.oecd.org/en/publications/ investing-in-medication-adherence-improves-health-outcomes-and-health-system-efficiency_8178962c-en.html [Accessed 2026-08-11]

100. Mash R, Christian C, Chigwanda RV. Alternative mechanisms for delivery of medication in South Africa: a scoping review. S Afr Fam Pract. Aug 24, 2001;63(1):e1-e8. [doi: 10.4102/safp.v63i1.5274]

52. Gackowski M, Jasińska-Stroschein M, Osmałek T, Waszyk-Nowaczyk M. Innovative approaches to enhance and measure medication adherence in chronic disease management: a review. Med Sci Monit. Jul 16, 2024;30:e944605. [doi: 10.12659/MSM.944605] [Medline: 39012851]

56. Miguel-Cruz A, Bohórquez AF, Parra PAA. What does the literature say about using electronic pillboxes for older adults? A systematic literature review. Disabil Rehabil Assistive Technol. Nov 2019;14(8):776-787. [doi: 10.1080/17483107.2018.1508514] [Medline: 30451543]

32. Mira JJ, Navarro I, Botella F, et al. A Spanish pillbox app for elderly patients taking multiple medications: randomized controlled trial. J Med Internet Res. Apr 4, 2014;16(4):e99. [doi: 10.2196/jmir.3269] [Medline: 24705022]

41. Balasubramanian GV, Beaney P, Chambers R. Digital personal assistants are smart ways for assistive technology to aid the health and wellbeing of patients and carers. BMC Geriatr. Nov 15, 2021;21(1):643. [doi: 10.1186/s12877-021- 02436-y] [Medline: 34781881]

43. Ahmad A, Chiu V, Arain MA. Users’ perceptions of an in-home electronic medication dispensing system: a qualitative study. Med Devices (Auckl). 2020;13:31-39. [doi: 10.2147/MDER.S241062] [Medline: 32104106]

46. Hincapie AL, Gupta V, Brown SA, Metzger AH. Exploring perceived barriers to medication adherence and the use of mobile technology in underserved patients with chronic conditions. J Pharm Pract. Apr 2019;32(2):147-153. [doi: 10.1177/0897190017744953] [Medline: 29212410]

47. LaValley SA, Brady LA, Nguyen M, Jacobs D. Family caregivers of older adults: strategies, tools, and technologies for medication management. J Gerontol Soc Work. 2023;66(2):291-302. [doi: 10.1080/01634372.2022.2100856] [Medline: 35850634]

38. Reidel K, Tamblyn R, Patel V, Huang A. Pilot study of an interactive voice response system to improve medication refill compliance. BMC Med Inf Decis Making. Oct 9, 2008;8:46. [doi: 10.1186/1472-6947-8-46] [Medline: 18845004]

106. Posadzki P, Mastellos N, Ryan R, et al. Automated telephone communication systems for preventive healthcare and management of long-term conditions. Cochrane Database Syst Rev. Dec 14, 2016;12(12):CD009921. [doi: 10.1002/ 14651858.CD009921.pub2] [Medline: 27960229]
