# Supplementary material for: Contexts and Mechanisms Related to the Efficacy of Digital Medication Adherence Interventions to Support Medication Adherence Among Adults With Chronic Diseases: Realist Review
Source: Interact J Med Res. 2026 Aug 28;15:e89803. doi: 10.2196/89803 (PMC13524365; doi:10.2196/89803)
Supplement: Supplementary file 5 [file ijmr-v15-e89803-s001.docx]

Presentation of the Medybox device and the aims of the co-creation workshops
